# Negative media portrayals of immigrants increase ingroup favoritism and hostile physiological and emotional reactions

**DOI:** 10.1038/s41598-021-95800-2

**Published:** 2021-08-12

**Authors:** Pierluigi Conzo, Giulia Fuochi, Laura Anfossi, Federica Spaccatini, Cristina Onesta Mosso

**Affiliations:** 1 Department of Economics and Statistics “Cognetti de Martiis”, University of Torino, Lungo Dora Siena 100A, 10153 Turin, Italy; 2 Collegio Carlo Alberto, Piazza Arbarello 8, 10122 Turin, Italy; 3 Department FISPPA - Applied Psychology, University of Padova, via Venezia 14, 35131 Padua, Italy; 4 Department of Chemistry, University of Torino, Via Giuria 5, 10125 Turin, Italy; 5 Department of Psychology, University of Milano Bicocca, Piazza dell’Ateneo Nuovo 1, 20126 Milan, Italy; 6 Department of Psychology, University of Torino, Via Giuseppe Verdi 10, 10124 Turin, Italy

**Keywords:** Human behaviour, Psychology and behaviour, Social evolution, Socioeconomic scenarios

## Abstract

Anti-immigration rhetoric in the mass media has intensified over the last two decades, potentially decreasing prosocial behavior and increasing outgroup hostility toward immigrants, and fostering ingroup favoritism toward natives. We aim to understand the effects of negative and positive discourses about immigration on prosociality at different levels of societal ethnic diversity. In two studies (student sample, nationally representative sample), we conduct a survey and a 3X3 between-subject experiment, including money-incentivized behavioral games measuring prosociality. We manipulate media representations of immigrants and the probability of interacting with immigrants (the latter measuring diversity). Results show that negative news affects prosociality as a function of the probability of interacting with immigrants. Negative portrayals increase altruism and trustworthiness in ethnically homogenous settings relative to unknown and ethnically-mixed contexts. These results are stronger for right-wing and high-prejudice respondents. Moreover, negative media portrayals of immigrants increase the testosterone-cortisol ratio, which is a proxy for proneness to social aggression. Negative news also increases outgroup-related perceived health risk, outgroup anxiety and outgroup threat less in ethnically-homogeneous contexts. Overall, negative portrayals of immigrants generate physiological and emotional hostility toward the outgroup, and ingroup favoritism in economic transactions, possibly determining efficiency losses in ethnically-diverse markets, relative to ethnically-homogeneous markets.

## Introduction

In the last two decades, political anti-immigration rhetoric has intensified in Western European countries and in the United States. Disputes over immigration issues (e.g., the Mediterranean refugee crisis, the Mexican border) have also become more frequent^1,2^. As a result, both traditional and social media—the latter often relying on unverified sources and socially provocative content^3^—have frequently framed immigrant people as a threat^1^. During the COVID-19 pandemic, anti-migrant rhetoric even made migrants—and sometimes foreign nationals more generally—into COVID-19 carriers^4^.

Negative depictions of immigration in public and media discourse increase prejudice and mistrust toward immigrants^5,6^ and the likelihood of anti-immigrant party voting^7^. Conversely, individuals exposed to news about immigration with politically-correct language tend to support less restrictive immigration and border security policies^8^. These phenomena can, from a social psychological perspective, be understood in the light of intergroup relations and intergroup conflict. A negative narrative on immigration fosters outgroup threat (from immigrants), and makes the differences and boundaries between the ingroup (natives) and the outgroup (immigrants) salient^9^. This strengthens ingroup identity and generates forms of ingroup favoritism, together with prejudice and discrimination toward the outgroup^10^.

Besides increasing prejudice and support for anti-immigrant leaders and policies, negative portrayals of immigrants in the mass media can heighten discrimination in socio-economic transactions. This includes buyer–seller, borrower–lender, or employer–employee interactions^11^. In trust games natives reciprocate, on average, the trust of immigrants less frequently than the trust of natives; this reciprocity gap increases if natives have negative attitudes toward ethnic diversity and immigration^12^. Low levels of trust, reciprocity, and altruism—that is, low levels of prosociality—undermine cooperation and the efficiency of economic exchanges^13,14^. As a result, anti-migrant rhetoric may have an unpredicted, perhaps unwanted, adverse economic impact. More specifically, negative media representations of immigrants may result in hostility and lower levels of prosociality in transactions with immigrants^5,6^; but negative media representations of immigrants may also result in higher levels of prosociality toward natives, due to ingroup favoritism or similar phenomena, such as parochial altruism^15^. Positive media representations of immigrants may, conversely, foster prosocial behavior toward immigrant transaction partners. Observing positively portrayed outgroup members in the media is a form of indirect positive intergroup contact^16^ and it is associated with more favorable attitudes toward the outgroup^5^. Therefore, positive portrayals of immigrants in the mass media could help close the immigrants-natives reciprocity gap found in previous research^12^.

However, partners are known only in fully informed interactions. Most of the time, individuals move through various markets characterized by imperfect information and incomplete contracts. In these contexts, natives can only estimate the probability of interaction with immigrants based on the available information on the ethnic diversity of these markets. There is a robust negative relationship between ethnic diversity and social trust, especially in local contexts^17^. Thus, when native people are exposed to negative portrayals of immigrants, their prosociality is likely to be lower in the case of markets or societies with medium or high ethnic diversity. This could be caused by both outgroup hostility^5,6^ and by the fact that ethnic diversity increases the uncertainty regarding shared social norms, including those related to prosociality^18^. Conversely, negative media representations of immigrants could increase prosociality when the probability of interaction with immigrants is low or close to zero (ethnically homogeneous markets), due to ingroup favoritism^10^. Positive media representations of immigrants may instead attenuate the negative relationship between societal ethnic diversity and prosociality^17^. If no information on potential transaction partners is available, a ‘veil of ignorance’ could promote more impartial decision-making^19^, helping to put aside mass-mediated portrayals of immigrants. Alternatively, people could fall back on their feelings and expectations on prospective transaction partners. If this is the case, the combination of negative media representations of immigrants and no information on transaction partners may raise suspicion and worry, with, consequently, decreased prosociality.

This paper aims at understanding the consequences of anti- or pro-immigration rhetoric in the media on prosocial economic behavior at different levels of societal ethnic diversity, depending on the probability of interaction between immigrants and natives. We also investigate the mechanisms and possible heterogeneity of these effects. Previous research has showed that negative portrayals of immigrants in the mass media increase prejudice^5–7^, and that people holding negative attitudes toward ethnic diversity are less prosocial toward immigrants^12^. Moreover, there is a robust negative association between ethnic diversity and social trust^17^. This paper investigates—as far as we know for the first time—the combined effect of media representations of immigrants and the level of ethnic diversity in a society or in a market. An endemic issue with non-experimental research in this area is that individuals choose media content and transaction partners. As such, confounding factors may impede the identification of the causal effects that media portrayals of immigrants have on prosociality. Confounding factors may also obscure how such causal effects change in terms of the ethnic diversity of expected interactions. By exogenously varying representations of immigration *and* the probability of being matched with immigrant partners, this paper sheds lights on how these two factors affect human prosociality in causal terms. This test is important because if negative portrayals of immigrants reduce prosociality in ethnically-diverse societies, they will also generate unexpected negative externalities for natives belonging to those societies. Moreover, negative portrayals of immigrants may increase prosociality in ethnically homogeneous contexts. If such effects are verified, in the long term anti-migrant rhetoric will produce a comparative disadvantage for ethnically-mixed societies.

In two studies (student sample, laboratory experiment; nationally representative sample, online experiment), we investigate the effects of media portrayals of immigrants and the ethnic diversity of potential transaction partners on prosocial choices (altruism, trust, and trustworthiness). We do so by using money-incentivized behavioral games. The list of potential transaction partners includes: immigrant and native names, or only native names, or unknown-origin names. We implement a 3 × 3 between-subject experiment, manipulating: (i) a 3-min television report portraying immigrants in a negative vs. positive way with no video (control condition); ii) the names of possible transaction partners: distinctively Italian names, *Italian list*; 50% Italian names and 50% names suggesting immigration background and mainly African origin, *mixed list* (representing an ethnically diverse society or market); ‘veil of ignorance’, *no list*. Then, respondents play money-incentivized behavioral games measuring altruism (dictator game, DG), trust and trustworthiness (trust game, TG).

Study 1 additionally explores the emotional and physiological reactions activated by the video. This is done through self-report measures of perceived outgroup threat^20^ and intergroup anxiety^21^ related to immigrants, and through the growth rate (before vs. after the video) of salivary testosterone-cortisol ratio, a proxy for proneness to social aggression^22^. These measures allow us to shed light on the mechanisms underlying the relationship between media portrayals of immigrants and prosocial behavior.

Study 2 replicates analyses in a nationally representative sample, and controls for a large set of individual characteristics, including pre-treatment self-report measures of social preferences^23^ and immigration attitudes (blatant and subtle prejudice;^24,25^). Importantly, in Study 2 we also test the presence of heterogeneity in treatment effects: more specifically, we investigate whether the combined effects of video and lists on prosocial choices are stronger for respondents with certain political orientations and attitudes toward immigrants. Moreover, in Study 2 we employ as outcome variables intergroup anxiety, perceived outgroup threat, and outgroup-related perceived health risk (during a pandemic). Only in Study 2 are outgroup threat and intergroup anxiety measured after both video and list manipulations.

## Results

### Study 1

Altruism and trust were measured in terms of the amount of money sent to the partner divided by the initial endowment, respectively in the DG and in the TG. Trustworthiness was measured in the TG and its models were conducted in the long-format dataset: trustees’ return decisions were distinct observations, nested within individuals (strategy method). Trustworthiness was computed as the amount returned divided by the hypothetical trustor’s transfer, multiplied by three and divided by 100 (to have a standardized measure).

We performed linear models with altruism, trust and trustworthiness predicted by age, gender, all conditions and their interactions (excluded categories: no video, no list). In all models, we employed robust standard errors (Huber and White correction); in the models for trustworthiness, standard errors were additionally clustered at the individual level to account for repeated choices within trustees. Full tables are reported in Supplementary Materials 1 (SM1). Figures report Tukey-adjusted pairwise comparisons of estimated marginal means, in which non-overlapping arrows indicate statistically significant differences. Since in laboratory studies there might be contextual factors affecting sessions in different ways, all the analyses of Study 1 were replicated with standard errors clustered at the session level (results reported in Supplementary Materials 3, SM3). Due to narrower confidence intervals, this second analysis confirmed the main results and showed more statistically significant effects. However, in the paper we report the more conservative estimates, i.e., the ones obtained with robust (Huber-White) standard errors.

**Figure 1:**
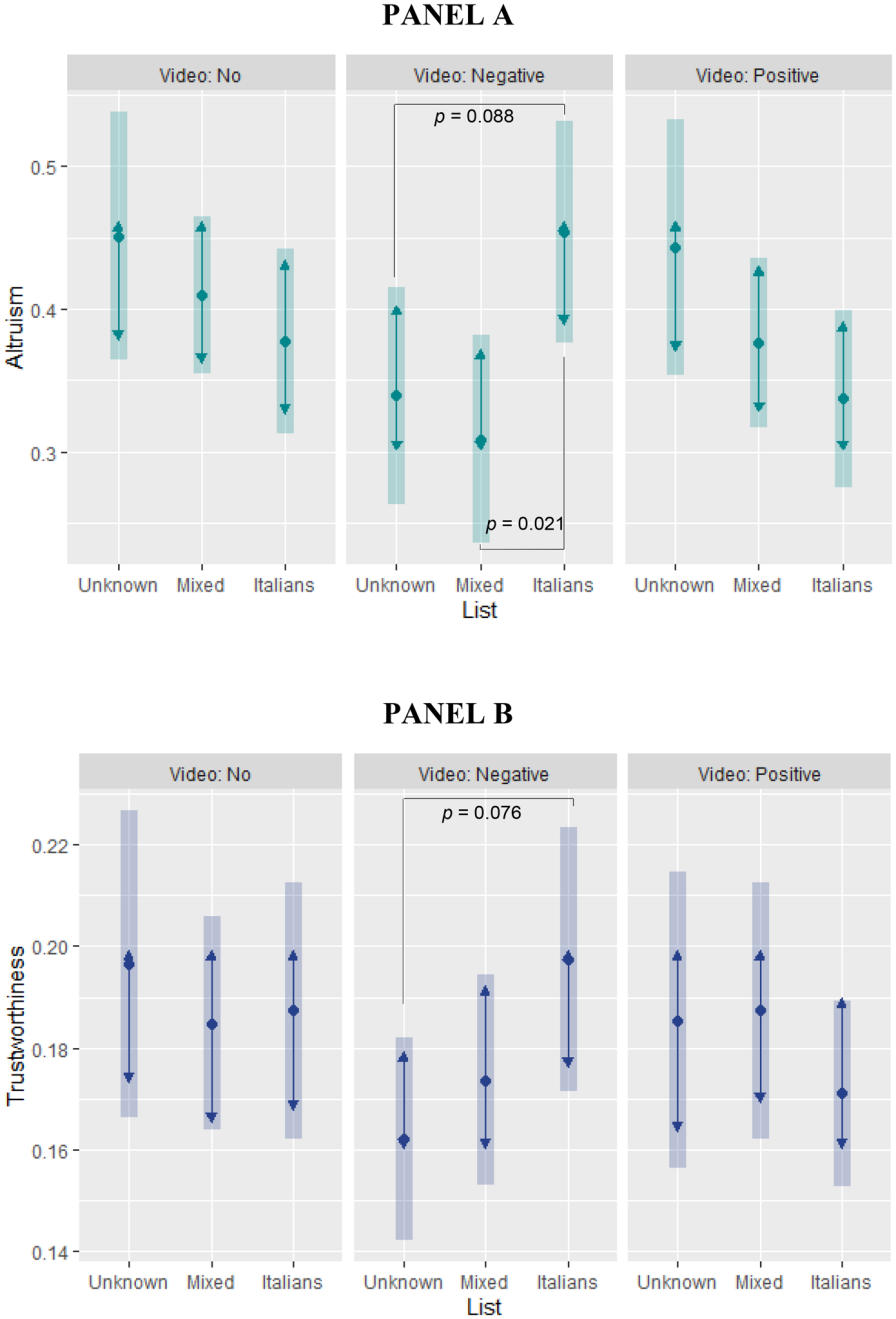
The effects of media portrayals of immigrants on prosociality (Study 1). Estimated marginal means (range 0–1) of altruism (**A**) and trustworthiness (**B**) by video and list conditions (Tukey adjustment for contrasts; bars: 95% confidence intervals; non-overlapping arrows: statistically significant pairwise comparison, *p* < 0.05). Altruism: money sent to the partner over initial endowment in dictator game; Trustworthiness: returned over received money in trust game, averaged across all hypothetical trustor’s transfers. Sample: Study 1 participants.

Results showed that altruism was negatively associated with negative video (b =  − 0.112, *p* = 0.058), and the interaction between negative video and Italian list was positive (b = 0.188, *p* = 0.016); apart from gender (being female), all other coefficients were not statistically significant (at *p* < 0.10). Pairwise comparisons (contrasts) of estimated marginal means (Fig. 1, panel A) showed that participants who watched the negative video were more altruistic in the Italian list than in the mixed list (+ 15 percentage points, *p* = 0.021) and no list (+ 11 percentage points, *p* = 0.088) conditions.

A similar pattern emerged in the model for trust, yet no statistically significant coefficients and pairwise comparisons of estimated marginal means emerged.

Trustworthiness was negatively associated with the negative video condition (b =  − 0.034, *p* = 0.063) and positively associated with the interaction between negative video and Italian list (b = 0.044, *p* = 0.085); all other coefficients were not statistically significant, except the hypothetical trustor’s transfer (b = 0.018, *p* < 0.001). Pairwise comparisons of estimated marginal means (Fig. 1, panel B) showed that participants who watched the negative video were more trustworthy in the Italian list than in no list (+ 4 percentage points, *p* = 0.076) condition. This is consistent with results on altruism. These results are from estimates employing the long-format version of the data, exploiting the full set of hypothetical strategies of the trustee and controlling for the trustor’s hypothetical transfers (see SM1).

To better understand the effect of the video net of the list conditions, i.e., under the veil of ignorance, we also computed pairwise comparisons of estimated marginal means in the no list condition. We did it for the altruism and trustworthiness linear models, in which the negative video has an effect. Results (available upon request) showed that in both models, none of the contrasts (no vs. negative video, no vs. positive video, negative vs. positive video) were statistically significant. These results, combined with the ones reported in Fig. 1, suggest that the negative video in Study 1 might have no effect by itself, whereas it generates a difference between the Italian list and the other conditions, especially the no list condition.

**Figure 2:**
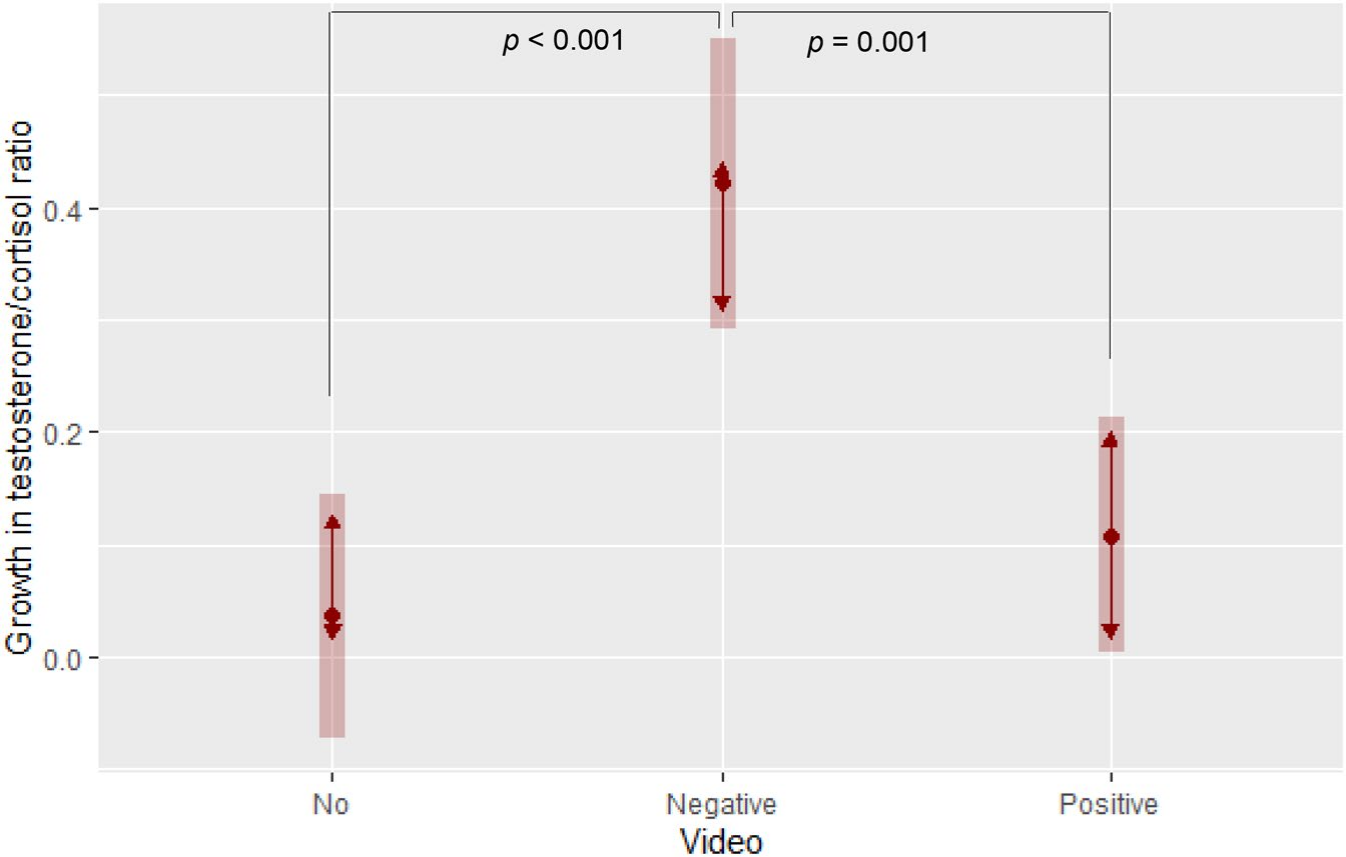
The physiological effects of media portrayals of immigrants (Study 1). Estimated marginal means (range 0–1) of testosterone-cortisol ratio growth rate by video conditions (Tukey adjustment for contrasts; bars: 95% confidence intervals; non-overlapping arrows: statistically significant pairwise comparison, *p* < 0.05). Sample: Study 1 participants.

To shed light on the emotional and physiological mechanisms activated by the video, we performed three linear models: intergroup anxiety, perceived outgroup threat (first extracted components of validated scales, asked between the video and the games), and growth in testosterone-cortisol ratio were predicted by age, gender and video conditions (also controlling for pre-video testosterone-cortisol ratio in the physiological model). The negative video was associated with increased perceived outgroup threat relative to no video (b = 0.540, *p* = 0.003), while no statistically significant effects of the positive video were found; no statistically significant effects were found for intergroup anxiety (b = 0.272, *p* = 0.251). Results were the same when anxiety and threat were measured by averaging items of each scale. In the physiological model (Fig. 2), the negative video increased the growth rate of testosterone-cortisol ratio (b = 0.385, *p* < 0.001) by 31 percentage points compared to positive video (*p* = 0.001) and 39 percentage points compared to no video (*p* < 0.001).

The effect of the video conditions on the results of behavioral games could be partly influenced by survey questions asked before the games. These had the potential to induce worries on immigration, or made the topic more salient than it would have otherwise been. Therefore, in Study 2 the same questions were asked after the games.

### Study 2

Altruism, trust and trustworthiness were measured as in Study 1; in this study, they were predicted by age, gender, income, education, marital status, all conditions and their interactions (excluded categories: no video, no list), duration of the survey, and pre-treatment social preferences or gift propensity. Full tables are reported in Supplementary Materials 1 (SM1).

**Figure 3:**
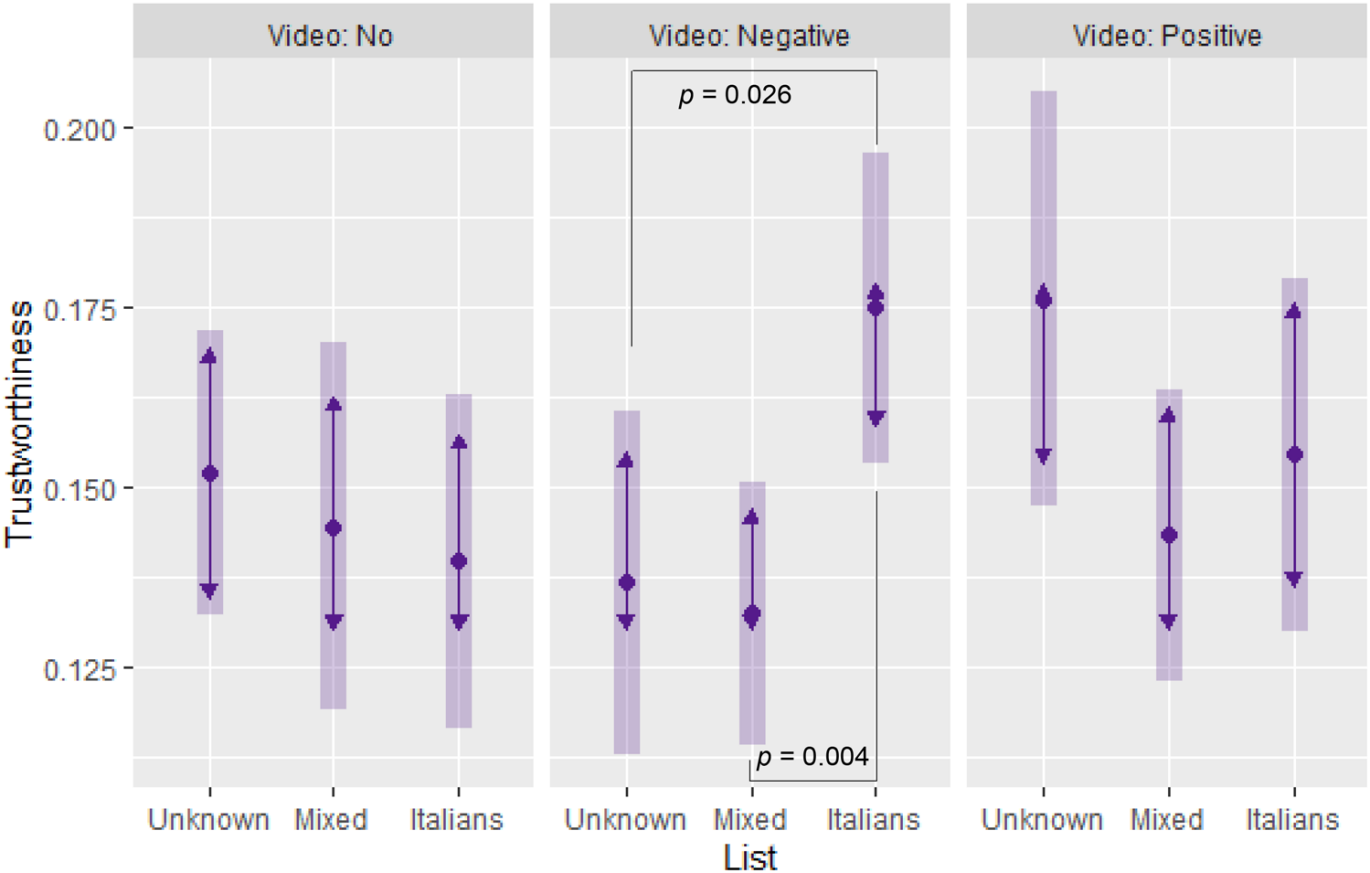
The effects of media portrayals of immigrants on trustworthiness (Study 2). Estimated marginal means (range 0–1) of trustworthiness by video and list conditions (Tukey adjustment for contrasts; bars: 95% confidence intervals; non-overlapping arrows: statistically significant pairwise comparison, *p* < 0.05). Trustworthiness: money returned over received in trust game, averaged across all hypothetical trustor’s transfers. Sample: Study 2 participants.

Results only partially confirmed the behavioral effects found in Study 1. More specifically, trustworthiness was positively associated with the interaction between the negative video and Italian list (b = 0.050, *p* = 0.015); no other coefficients were statistically significant, except social preferences (b = 0.008, *p* = 0.008) and the hypothetical trustor’s transfer (b = 0.014, *p* < 0.001). Pairwise comparisons of estimated marginal means (Fig. 3) showed that participants who watched the negative video were more trustworthy in the Italian list than in the mixed list (+ 4 percentage points, *p* = 0.004) and no list (+ 4 percentage points, *p* = 0.026) conditions. This was consistent with the results from Study 1.

To investigate the possible treatment heterogeneity of video and list conditions, linear models for altruism, trust and trustworthiness were performed again in sub-samples based on the respondent’s self-reported political orientation (left-wing vs. right-wing, respectively 0–5 and 6–10 in a 0–10 left–right bipolar scale), and on the respondent’s level of pre-treatment blatant and subtle prejudice toward immigrants. With political orientation, results showed that the combined effect of the negative video and the Italian list on trustworthiness was there for right-wing, but not for left-wing respondents. For right-wing respondents, trustworthiness was negatively associated with the negative video (b =  − 0.047, *p* = 0.050), and positively associated with the interaction between the negative video and the Italian list (b = 0.129, *p* < 0.001); no significant treatment effects on trustworthiness were found for left-wing respondents. A similar pattern emerged for trust: right-wing respondents’ trust was positively related to the interaction between the negative video and the Italian list (b = 0.266, *p* = 0.017), whereas the conditions had no statistically significant effects on the trust of left-wing respondents. As far as blatant and subtle prejudice toward immigrants were concerned, results showed that the combined effect of the negative video and the Italian list on trustworthiness emerged only for respondents with levels of prejudice above the median. In fact, trustworthiness was positively associated with the interaction between negative video and Italian list at high levels of blatant prejudice (b = 0.061, *p* = 0.027) and subtle prejudice (b = 0.066, *p* = 0.022). On the other hand, no significant treatment effects on trustworthiness were found for respondents with low levels of prejudice toward immigrants.

**Figure 4:**
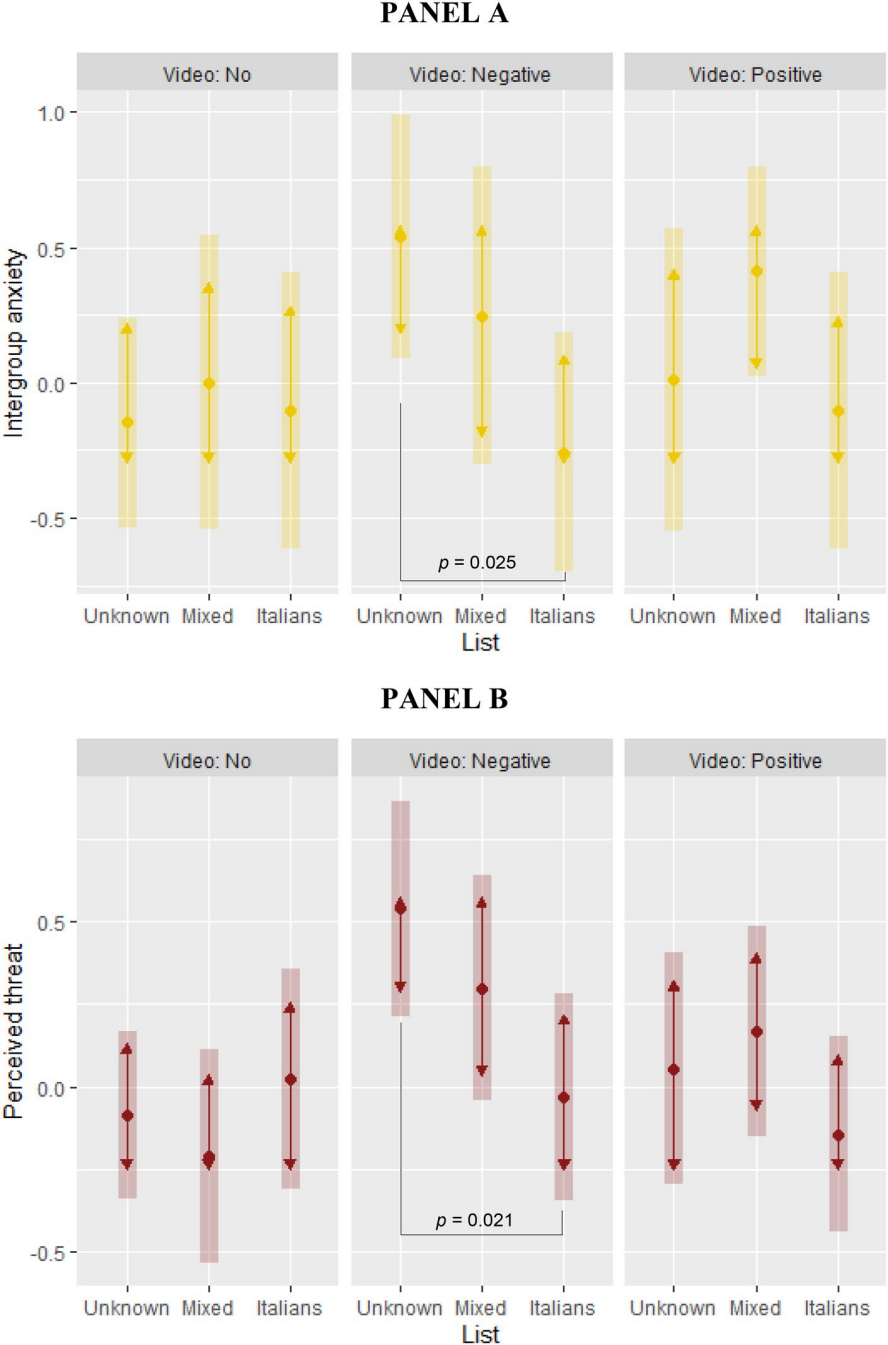
The effects of media portrayals of immigrants on intergroup anxiety and perceived outgroup threat (Study 2). Estimated marginal means of intergroup anxiety (**A**) and perceived outgroup threat (**B**) by video and list conditions (Tukey adjustment for contrasts; bars: 95% confidence intervals; non-overlapping arrows: statistically significant pairwise comparison, *p* < 0.05). Intergroup anxiety: first extracted component from a principal component analysis on answers to six questions measuring intergroup anxiety^22^. Perceived outgroup threat: first extracted component from a principal component analysis on answers to five questions measuring perceived outgroup threat^20^. Sample: Study 2 participants.

To test the emotional intergroup reactions to manipulations, we repeated the baseline linear models above with intergroup anxiety and perceived outgroup threat (the first extracted components of validated scales, asked after both the video and the games) as outcome variables. In these models, we also controlled for pre-treatment anti-immigration attitudes (first extracted factor from a pca on all items from blatant and subtle prejudice scales). Results showed that these two variables were positively associated with the negative video (b = 0.688, *p* = 0.015 for intergroup anxiety; b = 0.627, *p* = 0.001 for perceived outgroup threat), but negatively associated with the interaction between the negative video and the Italian list (b =  − 0.843, *p* = 0.058 for intergroup anxiety; b =  − 0.681, *p* = 0.021 for perceived outgroup threat). Among controls, pre-treatment immigration attitudes significantly predicted all outcomes (b = 0.289, *p* < 0.001 for intergroup anxiety; 0.614, *p* < 0.001 for perceived threat). Pairwise comparisons of estimated marginal means (Fig. 4, panels A-B) showed that participants who watched the negative video displayed lower intergroup anxiety and lower perceived threat in the Italian list than in the no list condition. This is consistent with the results for trustworthiness.

Study 2 was carried out in the year of the COVID-19 outbreak, during a period with relatively low levels of mortality and infections. To test whether immigrant portrayals also played a role in perceived health risk, we performed again the baseline linear model (controlling for pre-treatment immigration attitudes). We did so by using as outcome variable participants’ self-reported belief that immigrants represent a health risk for the population during a pandemic (asked after manipulations). Despite the potential health risks from immigration not being discussed in the video, which was prepared before the COVID-19 outbreak, perceived health risk was positively associated with the negative video condition (b = 0.651, *p* = 0.021). It was also negatively associated with the interaction between the negative video and Italian list conditions (b = − 0.948, *p* = 0.013). Pre-treatment immigration attitudes were the only statistically significant predictor (b = 1.198, *p* < 0.001).

## Discussion

In this paper, negative mass-mediated portrayals of immigrants strengthened altruism and trustworthiness in ethnically homogenous contexts; by this we mean contexts where respondents had zero probability of interacting with immigrant people, relative to ethnically-mixed and unknown contexts. The result for trustworthiness was supported by both studies, whereas the result for altruism emerged only in Study 1.

Results also indicated the possible mechanisms at play. Anti-immigration rhetoric increases negative emotional reactions related to immigrants. These include: perceived outgroup-related health risk; intergroup anxiety; outgroup threat; and aggressive physiological reactions, measured with the testosterone-cortisol ratio. However, as shown in Study 2, when individuals are exposed to negative portrayals of immigrants, interacting in a native-only society—compared to an ethnically diverse or an unknown context—reduces outgroup-related health risk, outgroup threat, and intergroup anxiety. Therefore, the most consistent finding is the interaction between negative video and Italian list, for multiple outcomes.

Overall, these results suggest that negative media representations of immigrants generate negative reactions at the emotional level, especially as shown in Study 1, and ingroup favoritism and parochial altruism^10,15^ at the behavioral level. On the one hand, anti-immigration rhetoric increases feelings of threat associated to immigrants, which worsen intergroup relations within society^9^. On the other hand, anti-immigration narratives generate differences in prosociality among contexts characterized by different levels of ethnic diversity: this creates a comparative advantage in markets or transactions with zero ethnic diversity compared to unknown or ethnically-mixed contexts. Consequently, ethnically-mixed contexts could lose mutually beneficial intergroup transactions.

Several mechanisms could explain this ingroup favoritism. First, the combination of anti-immigration narrative (negative video) and the awareness of being in an ethnically homogeneous society (Italian list) could have made ingroup identity more salient, thereby waking up the “us and them” dichotomy that is not salient in the two conditions taken separately (negative video, Italian list). According to social identity theory, a minimal sense of ingroup identity is enough to foster ingroup favoritism^10^, which is represented here by the higher levels of altruism and trustworthiness in an ingroup-only market compared to unknown or mixed ingroup-outgroup markets. Second, survey results suggest that when immigrants are portrayed in a negative way, individuals feel more at ease, i.e., they experience less anxiety and lower levels of threat, in interactions with only ingroup members, in this case Italians: this is compared to situations with a 50% or unknown probability of interacting with immigrants. This greater ease probably makes individuals more altruistic and trustworthy in ethnically homogenous contexts and represents another underlying mechanism for ingroup favoritism and parochial altruism^15^.

Lastly, heterogeneous treatment effects emerged: respondents who declared themselves more right-wing, or who endorsed higher levels of prejudice toward immigrants, were more prosocial only toward Italian transaction partners in response to negative portrayals of immigrants. On the contrary, the ingroup favoritism pattern was not present in left-wing and low-prejudice respondents. Therefore, mass-mediated anti-immigration rhetoric might have more influence on people who already hold negative expectations and beliefs in immigrants. This results in an extreme polarization of opinions, and possibly an echo-chamber effect^3^.

We acknowledge that the results of the two studies are not fully consistent. We have consistency across the two samples for three of the five outcomes (trust, trustworthiness, perceived outgroup threat but not for altruism and intergroup anxiety). However, participants in Study 2 seem less sensitive to video priming than participants (students) in Study 1. This could be due to several factors: (i) participants in Study 1 were more used to negative representations of immigrants because at the time of Study 2 (August 2020) there was much anti-immigration rhetoric in Italy; (ii) the negative video presented immigration as an economic, social and cultural problem, whereas at the time of Study 2 more salient themes related to immigrants were their free circulation and the associated health (COVID-19) consequences for Italians; (iii) unlike the participants in Study 1, respondents in Study 2 did not go through tasks involving the measurement of physiological parameters, which could have increased the stress and reactivity to stimuli in the participants of Study 1; (iv) as Study 2 was carried out in an uncontrolled environment, participants in that study might have gotten distracted more often while watching the video relative to students in a laboratory, in a separate workstation, isolated from any external stimulus. Another possible explanation to the partial consistency of the results is that the statistical power may not be enough to detect small behavioral effects, especially for interaction terms. Future studies could replicate our experiments in larger samples.

Interestingly, no significant effects were found for the mixed list condition. A possible explanation is that participants in the mixed list condition might have underestimated the probability of being matched with an immigrant—instead of a native—player. This explanation is perhaps particularly plausible in Study 1, in which participants saw other (native) participants in their lab session. Moreover, participants in the negative video condition underestimated the likelihood of being matched with an immigrant player more than participants in the other video conditions (see Supplementary Materials, SM2, section 4). The negative video might have made them believe that it was more unlikely that immigrants would be recruited. The underestimation of the probability of playing with immigrants might explain the unexpected absence of outgroup hostility in the combination of mixed list with the negative video.

We also acknowledge that no significant effects were found for the positive video condition. It is possible that participants paid more attention and were more responsive to the negative video—compared with the positive video—due to the so-called ‘negativity bias’, whereby negative news is more attractive than positive news^26^. So, the positive video may have resulted in a weaker priming compared to the negative video.

Despite these limitations, there are several take-aways from these results. First, generating a comparative advantage in markets or transactions with zero ethnic diversity compared to unknown or ethnically-mixed contexts may have negative economic and social consequences in the long run. If we imagine the homogeneous context and the diverse context as two neighborhoods of the same city, we can expect that the comparative advantage of the homogeneous context, in which there is more prosociality in transactions compared to the diverse context, will generate economic inequality and segregation within the city, thus in the overall society. Moreover, if media representations of immigrants do not match reality, it is likely that negative media portrayals of immigrants will make individuals underestimate the reciprocity of immigrant transaction partners, and overestimate the reciprocity of ingroup partners. In this way, mutually beneficial exchanges will be limited and there will be efficiency losses in trust-based transactions^27,28^.

Second, after negative portrayals of immigrants higher prosociality and lower threat and anxiety were found in the case of interactions with Italians only, compared to interactions with a mix of Italian and immigrant partners. As such anti-immigration rhetoric may represent a cost for ethnically-diverse societies. Importantly, such a cost is borne by everyone, both natives and non-natives. Leaders, politicians and influencers who choose to disseminate negative or biased information on minorities may want to consider the economic consequences of those actions in their societies, as ethnic diversity is increasing around the globe^29^.

Third, negative representations of immigrants physiologically predispose populations to hostility and heighten anxiety and a sense of threat, which may find outlets other than ingroup bias. For instance, there might be hate speech against minorities on social media or aggressive behavioral tendencies, with potentially dangerous social consequences.

Fourth, the ingroup favoritism we found in our results may explain the popularity and success of nationalist slogans like “Italians first” (#primagliitaliani) and “America first” within the anti-immigration campaigns of the last decade. Such campaigns can simultaneously generate negative emotional reactions against migrants and higher preference for, or greater ease associated with, native individuals.

This paper shed light on the socio-economic, emotional and physiological consequences of negative portrayals of immigrants in societies with varying degrees of ethnic diversity. Future research could usefully expand the understanding of the socio-economic consequences of xenophobic narratives in ethnically-diverse societies, testing the role of diverse information sources and of factors counteracting the behavioral and economic effects of biased news on immigration.

## Materials and methods

Games instructions, the on-line questionnaire, and details on physiological measures can be found at https://osf.io/jwv5a/. Full tables are available in SM1; additional analyses are reported in SM2.

The entire research was approved by the Ethical Committee of the University of Turin (protocol #55474). All participants delivered their informed consent before participating. All experiments were performed in accordance with relevant guidelines and regulations. A de-briefing session was carried out at the end of each study to clarify the aims of the research and the ad-hoc nature of the video. Before the data collection of Study 1, we performed power analyses with G*Power 3.1^30^. Considering the main effects of our predictors and the interactions between them, the required sample was 196 participants to observe a medium effect size (f = 0.25, α = 0.05, power = 0.80) of both interactions and main effects. All analyses were performed with the R^31^ packages *estimatr*^32^ and *emmeans*^33^.

### Study 1

Individuals (N = 384) were randomly extracted from 3942 students registered on the University on-line recruitment platform and assigned to 16 lab sessions. Participants who showed up (N = 375; final N = 350 because 25 were excluded as they had non-Italian parents) gave their informed consent and were randomly assigned to watching the negative television report, the positive television report, or nothing (control condition). Watching the video was the very first part of the experiment. The negative television report highlighted several negative aspects of immigration, such as its socio-economic cost and the threat to the cultural identity of natives. Conversely, the positive television report emphasized the positive aspects associated to immigration: in this case, immigrants were portrayed as a socio-economic resource for the receiving community, and as an opportunity to enrich the local culture. The reports’ effectiveness was confirmed by manipulation checks and by a preceding pilot study (see SM2): participants correctly identified the valence of immigrant portrayals, and understood whether immigrants were being portrayed as a cost/threat or as a resource/opportunity.

Participants were given two self-use oral swabs: the baseline sample was taken before the video, the second one after about 10 min (including video for non-control participants). After the video (or after the first saliva sample, for participants in the no-video condition), and before the second saliva sample, respondents answered questions measuring (i) perceived outgroup threat (five-item scale, 20) (ii) perceived intergroup anxiety (six-item scale, 21); (iii) other questions for another study with other research goals; and (iv) the perceived valence of the video.

Then, participants entered the behavioral games phase. They were randomly assigned to one of the three list conditions for subsequent money-incentivized behavioral tasks. To avoid experimenter-demand effects and unrealistic conditions, we did not employ a full immigrant-names list. To avoid deception, we employed names and decisions of same-session participants for the Italian and for the no list conditions, and of both session participants and immigrants from previously implemented experiments for the mixed list.

TG and DG appeared in randomized order. In the TG, the trustor was endowed with ten euros and decided how much to transfer to the trustee; the amount sent was tripled by the experimenter and was sent to the trustee, who decided how much to return to the trustor. Trustees made ten return decisions, one for each hypothetical trustor’s transfer (strategy method). In the DG, the sender was endowed with ten euros and decided how much to send to the receiver, who then took no action. Participants were informed about the structure of the games and payment before the tasks. They then played all roles playing both player roles in the TG and DG (the two games were randomly alternated). They finally took decisions in the Risky Decision task aimed at eliciting preferences towards risk (no significant treatment effects were found; results available in SM2). Game partners’ identity and game-specific payoffs were not revealed. After the games, participants were paid according to their payoff in one randomly selected game role (out of four), with one randomly selected game partner. The maximum (average) payoff was 20 (11.96) euros, including five euros as show-up fee.

### Study 2

For the sampling, programming, implementation and payments of Study 2, the polling company “Demetra” was contracted. A nationally representative (by age, gender, geographical origin) sample of Italian adults was recruited (N = 666) by the company and participated in an on-line survey lasting approximately 25 min. The sample only included native Italians; neither first- nor second-generation immigrants participated. Participants were randomly assigned to the same between-subject manipulations as in Study 1 (conditions: 3 video × 3 list).

After general instructions and informed consent, respondents answered (i) socio-demographic and media usage questions; (ii) a political orientation question (0–10 scale, from extreme left to extreme right); (iii) questions aimed at measuring gift propensity (used as a pre-treatment control in the linear models for altruism), social preferences (six items, aggregated by means of a pca, and used as a pre-treatment control in the linear models for trust and trustworthiness), and risk propensity (used as pre-treatment control in the linear models for risk propensity, not reported in the manuscript), all taken from^23^; (iv) two validated scales measuring prejudice toward immigrants, more specifically subtle prejudice (25, without positive emotions factor) and blatant prejudice^24^, which additionally included one item on perceived health risk associated with immigration (total 15 items, used in the pca for pre-treatment anti-immigration attitudes); (v) other questions for another study with other research goals; and (vi) an attention-check question.

Then, participants watched one of the three videos (randomly assigned), and entered the behavioral games (as in Study 1) immediately afterwards. Before playing, participants read the game instructions and were then shown a list containing either only Italian names (the Italian list) or half Italian and half immigrant names (the Mixed list); control participants received no list. Subjects in the list conditions were told that they could be matched with one of the listed players. The Italian list contained 23 (11 male and 12 female) names selected from the most popular names in Italy. The Mixed list conditions had 11 (five male and six female) names from the Italian list, plus 11 (six male and six female) non-Italian names chosen among the most popular ones in the country of origin of refugees (one male and one female name per nationality were selected from the top percentile of refugees’ nationality distribution in Italy). Selected names were kept constant within the list conditions, thereby minimizing potential gender, nationality and similarity effects.

After the games, participants answered a second attention-check question, and then to questions measuring: (i) the subjective probability of playing with an immigrant; (ii) perceived outgroup threat (five-item scale, 20); (iii) an item measuring the respondents’ belief that immigrants represent a health risk for the Italian population during a pandemic; (iv) intergroup anxiety (six-item scale, 21); (v) other questions for another study with other research goals; (vi) voting preferences and income. The survey ended with manipulation-check questions about the valence of the video and a debriefing screen, which informed participants that the matching with one of the other players in the list might not be consistent with the names shown in that list.

Overall, 606 participants were paid at the end of the survey by the company, which excluded 60 observations that did not meet their minimum data-quality standards (e.g., failed attention checks, fast or slow answers, missing values). The maximum (average) payoff was 15^7^ euros; a participation fee was added by the company.

Compared to highly controlled lab-experiments, on-line surveys are more subject to contextual factors affecting data quality. For this reason, data analysis was carried out on a total of 535 cases after data cleaning, which was based on the elimination of individuals who, in the order: (i) failed one of the two attention checks (N = 29); (ii) stayed on the video page less than the time of the video duration, namely 3 min (N = 25); (iii) fell above the ninetieth percentile of the time distribution on the video page (6′0.35″, N = 40); and (iv) took an implausibly short or long time to complete the survey considering the distribution of survey duration (N = 30), namely below the 2.5th percentile (no video: 8′; video: 12′0.01″) and above the 97.5th (no video: 53′0.06″; video: 57′0.96″) percentile of overall duration.

## Supplementary Information

Supplementary Information.
