# Supplementary material for: Negative media portrayals of immigrants increase ingroup favoritism and hostile physiological and emotional reactions
Source: Sci Rep. 2021 Aug 12;11:16407. doi: 10.1038/s41598-021-95800-2 (PMC8361166; doi:10.1038/s41598-021-95800-2)
Supplement: Supplementary file 1 — Supplementary Information. [file 41598_2021_95800_MOESM1_ESM.docx]

Full tables (SM1) and additional materials (SM2 and SM3) of the paper:

***Negative media portrayals of immigrants increase ingroup favoritism and hostile physiological and emotional reactions***

Pierluigi Conzo^a,b^*, Giulia Fuochi^c^, Laura Anfossi^d^, Federica Spaccatini^e^, Cristina Onesta Mosso^f^

^a^ Department of Economics and Statistics “Cognetti de Martiis”, University of Torino; Lungo Dora Siena 100A, 10153 Torino, Italy

^b^ Collegio Carlo Alberto; Piazza Arbarello 8, 10122 Torino, Italy

^c^ Department of Philosophy, Sociology, Education, and Applied Psychology, University of Padova; via Venezia 14, 35131 Padova, Italy

^d^ Department of Chemistry, University of Torino; Via Giuria 5, 10125 Torino, Italy

^e^ Department of Psychology, University of Milano “Bicocca”; Piazza dell’Ateneo Nuovo 1, 20126 Milano, Italy

^f^ Department of Psychology, University of Torino; Via Giuseppe Verdi 10, 10124 Torino, Italy

***Corresponding author**

Pierluigi Conzo, Department of Economics and Statistics “Cognetti de Martiis”, University of Torino; Lungodora Siena 100A, 10153 Torino, Italy. Phone number: +39 011 6703892. E-mail address: [pierluigi.conzo@unito.it](mailto:pierluigi.conzo@unito.it)

**Funding sources**: Fondazione Compagnia di San Paolo (Turin, Italy), project ‘InsideTrust’ (PI: P. Conzo); Fondazione Cassa di Risparmio di Torino (Turin, Italy), project ‘Closer to trust’ (PI: P. Conzo).

**Full tables (SM1)**

****** Study 1 ******

**Table S1. Summary statistics**

| Variable | Obs | Mean | Std. Dev. | Min | Max |
| --- | --- | --- | --- | --- | --- |
| OUTCOME VARIABLES: | | | | | |
| Trust | 346 | 0.485 | 0.291 | 0 | 1 |
| Giving | 348 | 0.384 | 0.229 | 0 | 1 |
| Trustworthiness (full trustee’s strategy) | 3,477 | 0.183 | 0.190 | 0 | 1 |
| Intergroup anxiety, pca –first extracted  factor | 350 | 2.4E-09 | 1.844 | -3.483 | 7.197 |
| Perceived outgroup threat, pca –first extracted  factor | 350 | 9.5E-09 | 1.678 | -2.056 | 7.163 |
| Cortisol, C (after video) | 326 | 0.264 | 0.093 | 0.07 | 0.817 |
| Testosterone, T (after video) | 327 | 105.460 | 75.064 | 7.32 | 737.09 |
| T/C (after video) | 323 | 416.984 | 291.374 | 32.247 | 2026.837 |
| Rate of growth of T/C | 323 | 0.131 | 0.653 | -0.871 | 2.898 |
| EXPERIMENTAL CONDITIONS: | | | | | |
| No list | 350 | 0.337 | 0.473 | 0 | 1 |
| Italian list | 350 | 0.320 | 0.467 | 0 | 1 |
| Mixed list | 350 | 0.343 | 0.475 | 0 | 1 |
| No video | 350 | 0.326 | 0.469 | 0 | 1 |
| Negative video | 350 | 0.349 | 0.477 | 0 | 1 |
| Positive video | 350 | 0.326 | 0.469 | 0 | 1 |
| CONTROL VARIABLES | | | | | |
| Age | 349 | 23.341 | 4.250 | 18 | 51 |
| Gender (being woman) | 349 | 0.625 | 0.485 | 0 | 1 |
| Cortisol, C (baseline) | 326 | 0.272 | 0.097 | 0.033 | 0.833 |
| Testosterone, T (baseline) | 327 | 109.492 | 76.722 | 11.310 | 786.660 |
| T/C (baseline) | 323 | 424.330 | 295.469 | 31.104 | 2115.152 |

**Table S2. The effects of video and list conditions on altruism: linear model with robust SEs**

|  | *b* | *95% CI(b)* | *SE(b)* | *p* |
| --- | --- | --- | --- | --- |
| Intercept | 0.387 | 0.241 – 0.534 | 0.075 | < 0.001 |
| Age | 0.004 | -0.001 – 0.009 | 0.003 | 0.148 |
| Gender (being woman) | -0.049 | -0.102 – 0.005 | 0.027 | 0.073 |
| Negative video | -0.112 | -0.227 – 0.004 | 0.059 | 0.058 |
| Positive video | -0.007 | -0.131 – 0.116 | 0.063 | 0.905 |
| Mixed list | -0.041 | -0.144 – 0.062 | 0.052 | 0.431 |
| Italian list | -0.074 | -0.182 – 0.035 | 0.055 | 0.183 |
| Negative video × Mixed list | 0.011 | -0.137 – 0.158 | 0.075 | 0.887 |
| Positive video × Mixed list | -0.026 | -0.173 – 0.121 | 0.075 | 0.730 |
| Negative video × Italian list | 0.188 | 0.036 – 0.341 | 0.078 | 0.016 |
| Positive video × Italian list | -0.033 | -0.184 – 0.119 | 0.077 | 0.673 |

*Note.* CI = Confidence Intervals; SE = Standard Errors.

**Table S3. The effects of video and list conditions on trust: linear model with robust SEs**

|  | *b* | *95% CI(b)* | *SE(b)* | *p* |
| --- | --- | --- | --- | --- |
| Intercept | 0.598 | 0.410 – 0.786 | 0.095 | < 0.001 |
| Age | -0.000 | -0.007 – 0.006 | 0.003 | 0.928 |
| Gender (being woman) | -0.114 | -0.181 – -0.047 | 0.034 | 0.001 |
| Negative video | -0.018 | -0.150 – 0.115 | 0.067 | 0.791 |
| Positive video | -0.034 | -0.173 – 0.104 | 0.070 | 0.626 |
| Mixed list | -0.060 | -0.185 – 0.066 | 0.064 | 0.350 |
| Italian list | -0.095 | -0.228 – 0.038 | 0.068 | 0.160 |
| Negative video × Mixed list | 0.036 | -0.144 – 0.216 | 0.092 | 0.693 |
| Positive video × Mixed list | 0.085 | -0.101 – 0.271 | 0.095 | 0.372 |
| Negative video × Italian list | 0.093 | -0.093 – 0.279 | 0.095 | 0.324 |
| Positive video × Italian list | 0.072 | -0.113 – 0.258 | 0.094 | 0.442 |

*Note.* CI = Confidence Intervals; SE = Standard Errors.

**Figure S1.** The effects of media portrayals of immigrants on Trust (Study 1)


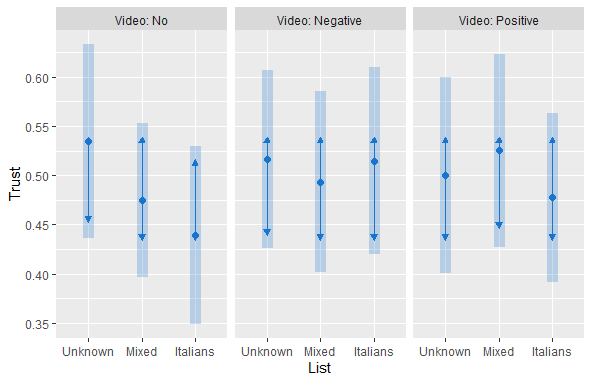


Estimated marginal means (range 0-1) of by video and list conditions (Tukey adjustment; bars: 95% confidence intervals; non-overlapping arrows: statistically significant pairwise comparison, *p* < .05). Sample: Study 1 participants.

**Table S4. The effects of video and list conditions on trustworthiness (full set of strategies): linear model with robust SEs**

|  | *b* | *95% CI(b)* | *SE(b)* | *p* |
| --- | --- | --- | --- | --- |
| Intercept | -0.103 | -0.156 – -0.050 | 0.027 | < 0.001 |
| Trustor’s hypothetical transfer | 0.018 | 0.017 – 0.019 | 0.000 | < 0.001 |
| Age | 0.000 | -0.002 – 0.002 | 0.001 | 0.734 |
| Gender (being woman) | -0.002 | -0.019 – 0.015 | 0.009 | 0.833 |
| Negative video | -0.034 | -0.071 – 0.002 | 0.018 | 0.063 |
| Positive video | -0.011 | -0.053 – 0.031 | 0.021 | 0.606 |
| Mixed list | -0.012 | -0.048 – 0.025 | 0.019 | 0.530 |
| Italian list | -0.009 | -0.049 – 0.030 | 0.020 | 0.648 |
| Negative video × Mixed list | 0.023 | -0.023 – 0.070 | 0.024 | 0.328 |
| Positive video × Mixed list | 0.014 | -0.040 – 0.067 | 0.027 | 0.618 |
| Negative video × Italian list | 0.044 | -0.006 – 0.095 | 0.026 | 0.085 |
| Positive video × Italian list | -0.005 | -0.058 – 0.047 | 0.027 | 0.844 |

*Note.* Robust standard errors clustered at the individual level. CI = Confidence Intervals; SE = Standard Errors. Dependent variable: [Trustee’s return transfer / (3 x Trustor’s hypothetical transfer)]/100. The dataset is in the “long” format, i.e. each Trustee’s return decision is a distinct observation (N = 3,467).

**Table S5. The effects of video conditions on growth rate of testosterone-cortisol ratio: linear model with robust SEs**

|  | *b* | *95% CI(b)* | *SE(b)* | *p* |
| --- | --- | --- | --- | --- |
| Intercept | 0.590 | 0.138 – 1.043 | 0.230 | 0.011 |
| Age | -0.001 | -0.015 – 0.013 | 0.007 | 0.917 |
| Gender (being woman) | -0.306 | -0.449 – -0.163 | 0.073 | < 0.001 |
| Baseline level | -0.001 | -0.001 – -0.001 | 0.000 | < 0.001 |
| Negative video | 0.385 | 0.215 – 0.555 | 0.086 | < 0.001 |
| Positive video | 0.072 | -0.074 – 0.217 | 0.074 | 0.334 |

*Note.* CI = Confidence Intervals; SE = Standard Errors.

**Table S6. The effects of video conditions on intergroup anxiety: linear model with robust SEs**

|  | *b* | *95% CI(b)* | *SE(b)* | *p* |
| --- | --- | --- | --- | --- |
| Intercept | 0.130 | -1.019 – 1.279 | 0.584 | 0.824 |
| Age | -0.013 | -0.058 – 0.031 | 0.023 | 0.557 |
| Gender (being woman) | 0.241 | -0.148 – 0.629 | 0.198 | 0.225 |
| Negative video | 0.272 | -0.193 – 0.736 | 0.236 | 0.251 |
| Positive video | -0.164 | -0.632 – 0.305 | 0.238 | 0.493 |

*Note.* CI = Confidence Intervals; SE = Standard Errors.

**Figure S2.** The effects of media portrayals of immigrants on intergroup anxiety (Study 1)


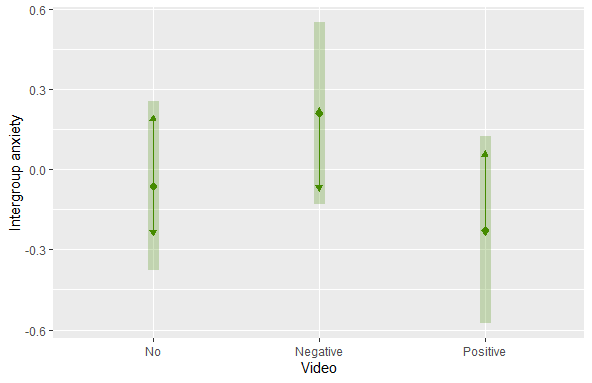


Estimated marginal means (range 0-1) of by video and list conditions (Tukey adjustment; bars: 95% confidence intervals; non-overlapping arrows: statistically significant pairwise comparison, *p* < .05). Sample: Study 1 participants.

**Table S7. The effects of video conditions on perceived outgroup threat: linear model with robust SEs**

|  | *b* | *95% CI(b)* | *SE(b)* | *p* |
| --- | --- | --- | --- | --- |
| Intercept | 0.377 | -0.504 – 1.258 | 0.448 | 0.401 |
| Age | -0.012 | -0.050 – 0.025 | 0.019 | 0.514 |
| Gender (being woman) | -0.540 | -0.894 – -0.186 | 0.180 | 0.003 |
| Negative video | 0.546 | 0.120 – 0.973 | 0.217 | 0.012 |
| Positive video | 0.210 | -0.189 – 0.610 | 0.203 | 0.301 |

*Note.* CI = Confidence Intervals; SE = Standard Errors

**Figure S3.** The effects of media portrayals of immigrants on perceived outgroup threat (Study 1)


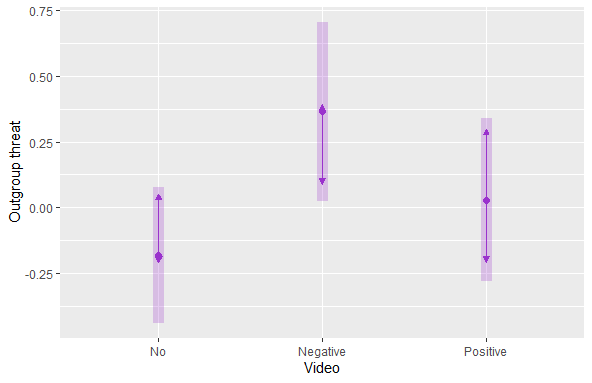


Estimated marginal means (range 0-1) of by video and list conditions (Tukey adjustment; bars: 95% confidence intervals; non-overlapping arrows: statistically significant pairwise comparison, *p* < .05). Sample: Study 1 participants.

****** Study 2 ******

**Table S1. Summary sample statistics**

| Variable | Obs | Mean | Std. Dev. | Min | Max |
| --- | --- | --- | --- | --- | --- |
| OUTCOME VARIABLES | | | | | |
| Giving | 535 | 0.375 | 0.234 | 0 | 1 |
| Trust | 535 | 0.445 | 0.259 | 0 | 1 |
| Trustworthiness (full trustee’s strategy) | 5,350 | 0.154 | 0.172 | 0 | 1 |
| Intergroup anxiety, pca – first extracted factor | 535 | 0.000 | 1.887 | -2.992 | 6.563 |
| Perceived outgroup threat, pca – first extracted factor | 535 | 0.000 | 1.951 | -3.442 | 3.607 |
| Outgroup-related health risk, pca –first extracted factor | 535 | 0.000 | 2.043 | -3.034 | 2.966 |
| EXPERIMENTAL CONDITIONS | | | | | |
| No video | 535 | 0.364 | 0.482 | 0 | 1 |
| Negative video | 535 | 0.318 | 0.466 | 0 | 1 |
| Positive video | 535 | 0.318 | 0.466 | 0 | 1 |
| No list | 535 | 0.331 | 0.471 | 0 | 1 |
| Italian list | 535 | 0.335 | 0.472 | 0 | 1 |
| Mixed list | 535 | 0.335 | 0.472 | 0 | 1 |
| MODERATORS | | | | | |
| Political orientation (pre-treatment) | 526 | 5.097 | 2.291 | 0 | 10 |
| Anti-immigration attitudes, pca –first extracted factor  (pre-treatment): |  |  |  |  |  |
| *blatant prejudice towards immigrants* | 535 | 0.000 | 2.005 | -5.101 | 4.038 |
| *subtle prejudice towards immigrants* | 535 | 0.000 | 1.888 | -4.626 | 3.710 |
| *subtle & blatant prejudice towards immigrants* | 535 | 0.000 | 2.557 | -6.800 | 5.476 |
| CONTROL VARIABLES | | | | | |
| Gender (being woman) | 535 | 0.516 | 0.500 | 0 | 1 |
| Age | 535 | 46.279 | 14.176 | 18 | 72 |
| Education: |  |  |  |  |  |
| *low* | 535 | 0.107 | 0.309 | 0 | 1 |
| *middle* | 535 | 0.536 | 0.499 | 0 | 1 |
| *high* | 535 | 0.357 | 0.480 | 0 | 1 |
| Married | 533 | 0.529 | 0.500 | 0 | 1 |
| Income class |  |  |  |  |  |
| *<500€* | 535 | 0.114 | 0.318 | 0 | 1 |
| *500€-1000€* | 535 | 0.157 | 0.364 | 0 | 1 |
| *1001€-1500€* | 535 | 0.250 | 0.434 | 0 | 1 |
| *1501€-2000€* | 535 | 0.178 | 0.383 | 0 | 1 |
| *2001€-2500€* | 535 | 0.084 | 0.278 | 0 | 1 |
| *2501€-3000€* | 535 | 0.041 | 0.199 | 0 | 1 |
| >*3000€* | 535 | 0.062 | 0.241 | 0 | 1 |
| *no answer* | 535 | 0.114 | 0.318 | 0 | 1 |
| Gift giving (pre-treatment): |  |  |  |  |  |
| *none* | 535 | 0.082 | 0.275 | 0 | 1 |
| *4* | 535 | 0.073 | 0.260 | 0 | 1 |
| *8* | 535 | 0.107 | 0.309 | 0 | 1 |
| *12* | 535 | 0.161 | 0.368 | 0 | 1 |
| *16* | 535 | 0.204 | 0.403 | 0 | 1 |
| *20* | 535 | 0.215 | 0.411 | 0 | 1 |
| *24* | 535 | 0.107 | 0.309 | 0 | 1 |
| *no answer* | 535 | 0.052 | 0.223 | 0 | 1 |
| Social preferences, pca –first extracted factor  (pre-treatment) | 535 | 0.000 | 1.371 | -4.799 | 2.856 |
| Lenght of survey (ln) | 535 | 3.105 | 0.365 | 2.100 | 4.058 |

**Table S2. The effects of video and list conditions on altruism: linear model with robust SEs**

|  | *b* | *95% CI(b)* | *SE(b)* | *p* |
| --- | --- | --- | --- | --- |
| Intercept | 0.334 | 0.123 – 0.546 | 0.108 | 0.002 |
| Negative video | 0.040 | -0.043 – 0.122 | 0.042 | 0.344 |
| Positive video | 0.069 | -0.027 – 0.166 | 0.049 | 0.160 |
| Mixed list | -0.007 | -0.085 – 0.070 | 0.039 | 0.851 |
| Italian list | 0.020 | -0.053 – 0.093 | 0.037 | 0.583 |
| Negative video × Mixed list | 0.032 | -0.086 – 0.151 | 0.060 | 0.593 |
| Positive video × Mixed list | -0.035 | -0.160 – 0.091 | 0.064 | 0.588 |
| Negative video × Italian list | -0.010 | -0.128 – 0.108 | 0.060 | 0.865 |
| Positive video × Italian list | -0.094 | -0.220 – 0.032 | 0.064 | 0.144 |
| Age | -0.001 | -0.002 – 0.001 | 0.001 | 0.463 |
| Gender (being woman) | -0.044 | -0.084 – -0.004 | 0.020 | 0.031 |
| Education: |  |  |  |  |
| [omitted = Low] |  |  |  |  |
| *Middle* | -0.033 | -0.088 – 0.022 | 0.028 | 0.237 |
| *High* | -0.015 | -0.077 – 0.047 | 0.032 | 0.635 |
| Married | 0.031 | -0.014 – 0.076 | 0.023 | 0.174 |
| Income class:  [omitted = <500€] |  |  |  |  |
| *500€-1000€* | -0.031 | -0.103 – 0.042 | 0.037 | 0.405 |
| *1001€-1500€* | -0.025 | -0.092 – 0.043 | 0.034 | 0.472 |
| *1501€-2000€* | -0.047 | -0.121 – 0.028 | 0.038 | 0.223 |
| *2001€-2500€* | 0.019 | -0.072 – 0.110 | 0.046 | 0.679 |
| *2501€-3000€* | -0.091 | -0.183 – -0.000 | 0.047 | 0.050 |
| *>3000€* | -0.047 | -0.154 – 0.060 | 0.055 | 0.389 |
| *no answer* | -0.120 | -0.197 – -0.042 | 0.040 | 0.003 |
| Gift giving (pre-treatment):  [omitted = no gift] |  |  |  |  |
| *4€* | -0.095 | -0.201 – 0.011 | 0.054 | 0.079 |
| *8€* | 0.020 | -0.082 – 0.122 | 0.052 | 0.701 |
| *12€* | 0.049 | -0.044 – 0.141 | 0.047 | 0.302 |
| *16€* | 0.073 | -0.021 – 0.166 | 0.048 | 0.127 |
| *20€* | 0.073 | -0.017 – 0.163 | 0.046 | 0.111 |
| *24€* | 0.126 | 0.020 – 0.232 | 0.054 | 0.020 |
| *No answer* | -0.105 | -0.216 – 0.007 | 0.057 | 0.067 |
| Lenght of survey (ln) | 0.023 | -0.034 – 0.079 | 0.029 | 0.432 |

*Note.* CI = Confidence Intervals; SE = Standard Errors.

**Figure 1.** The effects of media portrayals of immigrants on altruism (Study 2)


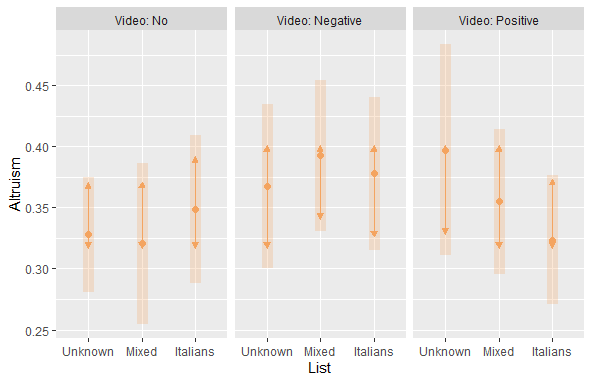


Estimated marginal means (range 0-1) of by video and list conditions (Tukey adjustment; bars: 95% confidence intervals; non-overlapping arrows: statistically significant pairwise comparison, *p* < .05). Sample: Study 2 participants

**Table S3. The effects of video and list conditions on trust: linear model with robust SEs**

|  | *b* | *95% CI(b)* | *SE(b)* | *p* |
| --- | --- | --- | --- | --- |
| Intercept | 0.060 | -0.164 – 0.283 | 0.114 | 0.600 |
| Negative video | -0.022 | -0.105 – 0.062 | 0.043 | 0.614 |
| Positive video | 0.012 | -0.093 – 0.116 | 0.053 | 0.825 |
| Mixed list | -0.013 | -0.096 – 0.069 | 0.042 | 0.751 |
| Italian list | 0.006 | -0.081 – 0.092 | 0.044 | 0.899 |
| Negative video × Mixed list | 0.074 | -0.055 – 0.202 | 0.065 | 0.260 |
| Positive video × Mixed list | 0.003 | -0.132 – 0.138 | 0.069 | 0.961 |
| Negative video × Italian list | 0.082 | -0.042 – 0.207 | 0.063 | 0.194 |
| Positive video × Italian list | -0.044 | -0.181 – 0.094 | 0.070 | 0.533 |
| Age | -0.001 | -0.003 – 0.001 | 0.001 | 0.339 |
| Gender (being woman) | -0.036 | -0.080 – 0.009 | 0.022 | 0.114 |
| Education: |  |  |  |  |
| [omitted = Low] | 0.017 | -0.050 – 0.084 | 0.034 | 0.624 |
| *Middle* | 0.042 | -0.030 – 0.115 | 0.037 | 0.248 |
| *High* | 0.052 | 0.003 – 0.101 | 0.025 | 0.039 |
| Married |  |  |  |  |
| Income class:  [omitted = <500€] | 0.006 | -0.074 – 0.085 | 0.040 | 0.885 |
| *500€-1000€* | 0.004 | -0.071 – 0.079 | 0.038 | 0.917 |
| *1001€-1500€* | -0.020 | -0.102 – 0.063 | 0.042 | 0.639 |
| *1501€-2000€* | 0.010 | -0.091 – 0.111 | 0.051 | 0.845 |
| *2001€-2500€* | -0.046 | -0.167 – 0.075 | 0.061 | 0.455 |
| *2501€-3000€* | -0.039 | -0.155 – 0.077 | 0.059 | 0.507 |
| *>3000€* | -0.066 | -0.160 – 0.029 | 0.048 | 0.175 |
| *no answer* | 0.129 | 0.062 – 0.196 | 0.034 | < 0.001 |
| Social preferences, pca (pre-treatment) | 0.023 | 0.005 – 0.041 | 0.009 | 0.011 |
| Lenght of survey (ln) | 0.129 | 0.062 – 0.196 | 0.034 | < 0.001 |

*Note.* CI = Confidence Intervals; SE = Standard Errors.

**Figure S2.** The effects of media portrayals of immigrants on trust (Study 2)


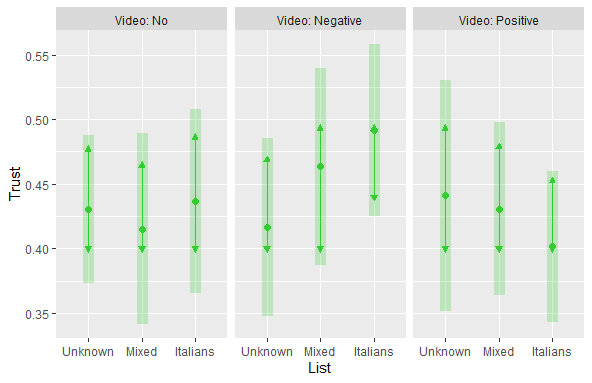


Estimated marginal means (range 0-1) of by video and list conditions (Tukey adjustment; bars: 95% confidence intervals; non-overlapping arrows: statistically significant pairwise comparison, *p* < .05). Sample: Study 2 participants.

**Table S4. The effects of video and list conditions on trustworthiness (full set of strategies): linear model with robust SEs**

|  | *b* | *95% CI(b)* | *SE(b)* | *p* |
| --- | --- | --- | --- | --- |
| Intercept | -0.124 | -0.203 - -0.045 | 0.040 | 0.002 |
| Trustor’s hypothetical transfer | 0.014 | 0.014 - 0.015 | 0.000 | < 0.001 |
| Negative video | -0.015 | -0.043 - 0.013 | 0.014 | 0.292 |
| Positive video | 0.024 | -0.010 - 0.059 | 0.018 | 0.170 |
| Mixed list | -0.007 | -0.037 - 0.023 | 0.015 | 0.630 |
| Italian list | -0.012 | -0.041 - 0.017 | 0.015 | 0.414 |
| Negative video × Mixed list | 0.003 | -0.037 - 0.043 | 0.020 | 0.880 |
| Positive video × Mixed list | -0.025 | -0.071 - 0.020 | 0.023 | 0.273 |
| Negative video × Italian list | 0.050 | 0.010 - 0.091 | 0.021 | 0.015 |
| Positive video × Italian list | -0.009 | -0.057 - 0.038 | 0.024 | 0.697 |
| Age | -0.000 | -0.001 - 0.000 | 0.000 | 0.419 |
| Gender (being woman) | -0.011 | -0.026 - 0.004 | 0.008 | 0.161 |
| Education:  [omitted = Low] |  |  |  |  |
| *Middle* | 0.004 | -0.022 - 0.030 | 0.013 | 0.736 |
| *High* | 0.012 | -0.015 - 0.039 | 0.014 | 0.389 |
| Married | 0.007 | -0.010 - 0.024 | 0.009 | 0.411 |
| Income class:  [omitted = <500€] |  |  |  |  |
| *500€-1000€* | 0.009 | -0.019 - 0.037 | 0.014 | 0.535 |
| *1001€-1500€* | -0.003 | -0.028 - 0.023 | 0.013 | 0.841 |
| *1501€-2000€* | -0.000 | -0.029 - 0.029 | 0.015 | 0.986 |
| *2001€-2500€* | -0.004 | -0.039 - 0.031 | 0.018 | 0.810 |
| *2501€-3000€* | -0.027 | -0.070 - 0.016 | 0.022 | 0.224 |
| *>3000€* | -0.014 | -0.054 - 0.026 | 0.021 | 0.501 |
| *no answer* | -0.009 | -0.036 - 0.018 | 0.014 | 0.511 |
| Social preferences, pca (pre-treatment) | 0.008 | 0.002 - 0.014 | 0.003 | 0.008 |
| Lenght of survey (ln) | 0.017 | -0.004 - 0.039 | 0.011 | 0.119 |

*Note.* CI = Confidence Intervals; SE = Standard Errors. Dependent variable: [Trustee’s return transfer / (3 x Trustor’s hypothetical transfer)]/100. The dataset is in the “long” format, i.e. each Trustee’s return decision is a distinct observation (N = 5,330).

**Table S5a. The partisan effects of video and list conditions on altruism: linear model with robust SEs**

|  | *Left-wing respondents*  *(≤ 5 in self-declared political orientation)* | | | | *Right-wing respondents*  *(> 5 in self-declared political orientation)* | | | |
| --- | --- | --- | --- | --- | --- | --- | --- | --- |
|  | *b* | *95% CI(b)* | *SE(b)* | *p* | *b* | *95% CI(b)* | *SE(b)* | *p* |
| Intercept | 0.370 | 0.094 – 0.647 | 0.141 | 0.009 | 0.261 | -0.125 – 0.646 | 0.195 | 0.184 |
| Negative video | 0.027 | -0.083 – 0.138 | 0.056 | 0.628 | 0.079 | -0.055 – 0.212 | 0.067 | 0.246 |
| Positive video | 0.069 | -0.064 – 0.202 | 0.068 | 0.310 | 0.072 | -0.066 – 0.210 | 0.070 | 0.304 |
| Mixed list | -0.115 | -0.207 – -0.023 | 0.047 | 0.014 | 0.116 | -0.012 – 0.244 | 0.065 | 0.076 |
| Italian list | 0.001 | -0.101 – 0.103 | 0.052 | 0.983 | 0.050 | -0.071 – 0.172 | 0.062 | 0.414 |
| Negative video × Mixed list | 0.127 | -0.022 – 0.275 | 0.076 | 0.095 | -0.109 | -0.301 – 0.082 | 0.097 | 0.262 |
| Positive video × Mixed list | 0.063 | -0.103 – 0.229 | 0.084 | 0.454 | -0.150 | -0.331 – 0.031 | 0.092 | 0.103 |
| Negative video × Italian list | 0.005 | -0.152 – 0.162 | 0.080 | 0.951 | -0.044 | -0.244 – 0.155 | 0.101 | 0.663 |
| Positive video × Italian list | -0.106 | -0.279 – 0.067 | 0.088 | 0.228 | -0.114 | -0.302 – 0.073 | 0.095 | 0.231 |
| Age | -0.001 | -0.003 – 0.001 | 0.001 | 0.579 | -0.001 | -0.004 – 0.002 | 0.002 | 0.540 |
| Gender (being woman) | -0.062 | -0.113 – -0.010 | 0.026 | 0.019 | -0.053 | -0.113 – 0.007 | 0.031 | 0.085 |
| Education:  [omitted = Low] |  |  |  |  |  |  |  |  |
| *Middle* | -0.059 | -0.132 – 0.013 | 0.037 | 0.109 | 0.026 | -0.061 – 0.114 | 0.044 | 0.552 |
| *High* | -0.050 | -0.133 – 0.033 | 0.042 | 0.236 | 0.037 | -0.065 – 0.139 | 0.052 | 0.473 |
| Married | 0.036 | -0.026 – 0.098 | 0.032 | 0.259 | 0.028 | -0.040 – 0.097 | 0.035 | 0.415 |
| Income class:  [omitted = <500€] |  |  |  |  |  |  |  |  |
| *500€-1000€* | -0.056 | -0.142 – 0.030 | 0.044 | 0.200 | 0.070 | -0.059 – 0.200 | 0.066 | 0.285 |
| *1001€-1500€* | -0.038 | -0.127 – 0.052 | 0.045 | 0.406 | 0.005 | -0.101 – 0.111 | 0.054 | 0.927 |
| *1501€-2000€* | -0.076 | -0.171 – 0.018 | 0.048 | 0.114 | 0.000 | -0.121 – 0.121 | 0.062 | 1.000 |
| *2001€-2500€* | 0.040 | -0.085 – 0.164 | 0.063 | 0.532 | 0.024 | -0.100 – 0.148 | 0.063 | 0.699 |
| *2501€-3000€* | -0.156 | -0.269 – -0.042 | 0.058 | 0.008 | -0.028 | -0.180 – 0.124 | 0.077 | 0.719 |
| *>3000€* | -0.095 | -0.212 – 0.022 | 0.059 | 0.112 | -0.036 | -0.225 – 0.153 | 0.096 | 0.709 |
| *no answer* | -0.121 | -0.220 – -0.021 | 0.051 | 0.018 | -0.076 | -0.201 – 0.049 | 0.063 | 0.230 |
| Gift giving (pre-treatment):  [omitted = no gift] |  |  |  |  |  |  |  |  |
| *4€* | -0.172 | -0.301 – -0.043 | 0.066 | 0.009 | 0.108 | -0.048 – 0.264 | 0.079 | 0.173 |
| *8€* | -0.037 | -0.174 – 0.100 | 0.070 | 0.592 | 0.146 | 0.009 – 0.283 | 0.069 | 0.037 |
| *12€* | -0.015 | -0.131 – 0.101 | 0.059 | 0.797 | 0.178 | 0.047 – 0.309 | 0.066 | 0.008 |
| *16€* | -0.028 | -0.147 – 0.091 | 0.060 | 0.648 | 0.266 | 0.137 – 0.394 | 0.065 | < 0.001 |
| *20€* | -0.023 | -0.133 – 0.087 | 0.056 | 0.684 | 0.257 | 0.128 – 0.386 | 0.065 | < 0.001 |
| *24€* | 0.079 | -0.058 – 0.217 | 0.070 | 0.256 | 0.265 | 0.117 – 0.414 | 0.075 | 0.001 |
| *No answer* | -0.167 | -0.334 – -0.000 | 0.085 | 0.049 | 0.066 | -0.068 – 0.199 | 0.068 | 0.332 |
| Lenght of survey (ln) | 0.060 | -0.015 – 0.136 | 0.038 | 0.117 | -0.042 | -0.140 – 0.055 | 0.050 | 0.394 |

*Note.* CI = Confidence Intervals; SE = Standard Errors

**Table S5b. The partisan effects of video and list conditions on trust: linear model with robust SEs**

|  | *Left-wing respondents*  *(≤ 5 in self-declared political orientation)* | | | | *Right-wing respondents*  *(> 5 in self-declared political orientation)* | | | |
| --- | --- | --- | --- | --- | --- | --- | --- | --- |
|  | *b* | *95% CI(b)* | *SE(b)* | *p* | *b* | *95% CI(b)* | *SE(b)* | *p* |
| Intercept | 0.032 | -0.250 – 0.313 | 0.143 | 0.825 | 0.207 | -0.217 – 0.631 | 0.215 | 0.336 |
| Negative video | 0.040 | -0.066 – 0.146 | 0.054 | 0.459 | -0.060 | -0.192 – 0.072 | 0.067 | 0.370 |
| Positive video | 0.082 | -0.056 – 0.221 | 0.071 | 0.244 | -0.054 | -0.214 – 0.106 | 0.081 | 0.508 |
| Mixed list | -0.083 | -0.182 – 0.016 | 0.050 | 0.100 | 0.061 | -0.092 – 0.214 | 0.078 | 0.430 |
| Italian list | 0.046 | -0.072 – 0.164 | 0.060 | 0.443 | -0.051 | -0.202 – 0.100 | 0.077 | 0.507 |
| Negative video × Mixed list | 0.081 | -0.079 – 0.242 | 0.082 | 0.320 | 0.023 | -0.175 – 0.221 | 0.100 | 0.817 |
| Positive video × Mixed list | -0.002 | -0.178 – 0.173 | 0.089 | 0.980 | -0.002 | -0.213 – 0.208 | 0.107 | 0.982 |
| Negative video × Italian list | -0.055 | -0.216 – 0.107 | 0.082 | 0.506 | 0.266 | 0.048 – 0.484 | 0.111 | 0.017 |
| Positive video × Italian list | -0.154 | -0.339 – 0.031 | 0.094 | 0.102 | 0.053 | -0.155 – 0.262 | 0.106 | 0.613 |
| Age | -0.000 | -0.003 – 0.002 | 0.001 | 0.793 | -0.002 | -0.005 – 0.001 | 0.002 | 0.210 |
| Gender (being woman) | -0.043 | -0.097 – 0.012 | 0.028 | 0.124 | -0.036 | -0.114 – 0.041 | 0.039 | 0.354 |
| Education:  [omitted = Low] |  |  |  |  |  |  |  |  |
| *Middle* | 0.021 | -0.064 – 0.106 | 0.043 | 0.626 | 0.003 | -0.106 – 0.112 | 0.055 | 0.961 |
| *High* | 0.065 | -0.024 – 0.154 | 0.045 | 0.151 | -0.013 | -0.137 – 0.111 | 0.063 | 0.838 |
| Married | 0.043 | -0.023 – 0.110 | 0.034 | 0.204 | 0.072 | -0.003 – 0.146 | 0.038 | 0.059 |
| Income class:  [omitted = <500€] |  |  |  |  |  |  |  |  |
| *500€-1000€* | 0.018 | -0.080 – 0.117 | 0.050 | 0.717 | -0.005 | -0.145 – 0.135 | 0.071 | 0.944 |
| *1001€-1500€* | 0.008 | -0.082 – 0.099 | 0.046 | 0.854 | -0.015 | -0.154 – 0.125 | 0.071 | 0.835 |
| *1501€-2000€* | 0.006 | -0.096 – 0.109 | 0.052 | 0.902 | -0.076 | -0.216 – 0.065 | 0.071 | 0.289 |
| *2001€-2500€* | 0.030 | -0.088 – 0.147 | 0.060 | 0.620 | -0.039 | -0.235 – 0.157 | 0.100 | 0.695 |
| *2501€-3000€* | -0.055 | -0.203 – 0.094 | 0.075 | 0.467 | -0.063 | -0.280 – 0.154 | 0.110 | 0.568 |
| *>3000€* | -0.083 | -0.219 – 0.052 | 0.069 | 0.228 | -0.061 | -0.277 – 0.155 | 0.110 | 0.578 |
| *no answer* | -0.019 | -0.143 – 0.104 | 0.063 | 0.760 | -0.124 | -0.279 – 0.032 | 0.079 | 0.118 |
| Social preferences, pca (pre-treatment) | 0.023 | -0.000 – 0.047 | 0.012 | 0.055 | 0.021 | -0.007 – 0.048 | 0.014 | 0.141 |
| Lenght of survey (ln) | 0.132 | 0.045 – 0.218 | 0.044 | 0.003 | 0.100 | -0.014 – 0.214 | 0.058 | 0.086 |

*Note.* CI = Confidence Intervals; SE = Standard Errors.

**Table S5c. The partisan effects of video and list conditions on trustworthiness: linear model with robust SEs**

|  | *Left-wing respondents*  *(≤ 5 in self-declared political orientation)* | | | | *Right-wing respondents*  *(> 5 in self-declared political orientation)* | | | |
| --- | --- | --- | --- | --- | --- | --- | --- | --- |
|  | *b* | *95% CI(b)* | *SE(b)* | *p* | *b* | *95% CI(b)* | *SE(b)* | *p* |
| Intercept | -0.163 | -0.253 – -0.073 | 0.046 | <0.001 | -0.060 | -0.212 – 0.093 | 0.077 | 0.441 |
| Trustor’s hypothetical transfer | 0.014 | 0.013 – 0.015 | 0.000 | <0.001 | 0.015 | 0.014 – 0.016 | 0.001 | <0.001 |
| Negative video | 0.006 | -0.028 – 0.041 | 0.018 | 0.719 | -0.047 | -0.094 – 0.000 | 0.024 | 0.050 |
| Positive video | 0.040 | -0.004 – 0.084 | 0.022 | 0.076 | -0.004 | -0.061 – 0.054 | 0.029 | 0.901 |
| Mixed list | -0.007 | -0.043 – 0.029 | 0.018 | 0.717 | -0.020 | -0.072 – 0.032 | 0.026 | 0.457 |
| Italian list | 0.003 | -0.033 – 0.039 | 0.018 | 0.878 | -0.029 | -0.080 – 0.023 | 0.026 | 0.273 |
| Negative video × Mixed list | -0.018 | -0.068 – 0.032 | 0.025 | 0.472 | 0.037 | -0.030 – 0.103 | 0.034 | 0.279 |
| Positive video × Mixed list | -0.038 | -0.097 – 0.021 | 0.030 | 0.204 | 0.008 | -0.066 – 0.082 | 0.037 | 0.828 |
| Negative video × Italian list | 0.002 | -0.048 – 0.051 | 0.025 | 0.945 | 0.129 | 0.059 – 0.199 | 0.036 | <0.001 |
| Positive video × Italian list | -0.050 | -0.110 – 0.010 | 0.030 | 0.100 | 0.057 | -0.024 – 0.137 | 0.041 | 0.166 |
| Age | -0.000 | -0.001 – 0.000 | 0.000 | 0.467 | -0.000 | -0.001 – 0.001 | 0.001 | 0.656 |
| Gender (being woman) | -0.006 | -0.024 – 0.012 | 0.009 | 0.494 | -0.008 | -0.035 – 0.019 | 0.014 | 0.578 |
| Education:  [omitted = Low] |  |  |  |  |  |  |  |  |
| *Middle* | 0.007 | -0.023 – 0.038 | 0.015 | 0.630 | -0.004 | -0.052 – 0.044 | 0.024 | 0.861 |
| *High* | 0.010 | -0.022 – 0.041 | 0.016 | 0.543 | 0.017 | -0.033 – 0.067 | 0.025 | 0.504 |
| Married | 0.011 | -0.010 – 0.031 | 0.011 | 0.319 | -0.000 | -0.030 – 0.030 | 0.015 | 0.991 |
| Income class:  [omitted = <500€] |  |  |  |  |  |  |  |  |
| *500€-1000€* | 0.016 | -0.016 – 0.049 | 0.016 | 0.312 | -0.009 | -0.063 – 0.044 | 0.027 | 0.730 |
| *1001€-1500€* | 0.002 | -0.025 – 0.030 | 0.014 | 0.861 | -0.017 | -0.067 – 0.033 | 0.025 | 0.498 |
| *1501€-2000€* | 0.005 | -0.027 – 0.037 | 0.016 | 0.756 | -0.011 | -0.069 – 0.046 | 0.029 | 0.695 |
| *2001€-2500€* | 0.033 | -0.008 – 0.073 | 0.021 | 0.115 | -0.065 | -0.126 – -0.003 | 0.031 | 0.038 |
| *2501€-3000€* | -0.014 | -0.070 – 0.042 | 0.028 | 0.632 | -0.063 | -0.139 – 0.012 | 0.038 | 0.098 |
| *>3000€* | -0.012 | -0.063 – 0.039 | 0.026 | 0.649 | -0.031 | -0.105 – 0.043 | 0.037 | 0.405 |
| *no answer* | -0.005 | -0.035 – 0.026 | 0.015 | 0.770 | -0.027 | -0.074 – 0.021 | 0.024 | 0.275 |
| Social preferences, pca (pre-treatment) | 0.010 | 0.004 – 0.017 | 0.003 | 0.002 | 0.005 | -0.005 – 0.014 | 0.005 | 0.349 |
| Lenght of survey (ln) | 0.026 | 0.000 – 0.052 | 0.013 | 0.046 | 0.004 | -0.035 – 0.044 | 0.020 | 0.824 |

CI = Confidence Intervals; SE = Standard Errors. Dependent variable: [Trustee’s return transfer / (3 x Trustor’s hypothetical transfer)]/100. The dataset is in the “long” format, i.e. each Trustee’s return decision is a distinct observation.

**Table S6a. The effects of video and list conditions on altruism by levels of *blatant* prejudice towards immigrants: linear model with robust SEs**

|  | *≤ median blatant prejudice towards immigrants* | | | | *> median blatant prejudice towards immigrants* | | | |
| --- | --- | --- | --- | --- | --- | --- | --- | --- |
|  | *b* | *95% CI(b)* | *SE(b)* | *p* | *b* | *95% CI(b)* | *SE(b)* | *p* |
| Intercept | 0.438 | 0.132 – 0.744 | 0.155 | 0.005 | 0.281 | -0.015 - 0.577 | 0.150 | 0.063 |
| Negative video | -0.016 | -0.140 – 0.109 | 0.063 | 0.804 | 0.075 | -0.045 - 0.196 | 0.061 | 0.220 |
| Positive video | 0.071 | -0.078 – 0.220 | 0.076 | 0.350 | 0.016 | -0.120 – 0.151 | 0.069 | 0.820 |
| Mixed list | -0.065 | -0.180 – 0.050 | 0.058 | 0.270 | 0.040 | -0.076 – 0.155 | 0.059 | 0.499 |
| Italian list | -0.014 | -0.139 – 0.110 | 0.063 | 0.820 | 0.035 | -0.059 – 0.130 | 0.048 | 0.461 |
| Negative video × Mixed list | 0.121 | -0.062 – 0.304 | 0.093 | 0.193 | -0.039 | -0.212 – 0.134 | 0.088 | 0.657 |
| Positive video × Mixed list | -0.002 | -0.190 – 0.186 | 0.096 | 0.983 | 0.004 | -0.180 – 0.188 | 0.094 | 0.966 |
| Negative video × Italian list | 0.050 | -0.135 – 0.235 | 0.094 | 0.598 | -0.052 | -0.223 – 0.119 | 0.087 | 0.552 |
| Positive video × Italian list | -0.066 | -0.260 – 0.129 | 0.099 | 0.507 | -0.080 | -0.250 – 0.089 | 0.086 | 0.350 |
| Age | -0.002 | -0.004 – 0.000 | 0.001 | 0.054 | 0.002 | -0.001 – 0.004 | 0.001 | 0.186 |
| Gender (being woman) | -0.071 | -0.132 – -0.011 | 0.031 | 0.021 | -0.030 | -0.085 – 0.026 | 0.028 | 0.293 |
| Education:  [omitted = Low] |  |  |  |  |  |  |  |  |
| *Middle* | -0.016 | -0.087 – 0.055 | 0.036 | 0.658 | -0.050 | -0.130 – 0.030 | 0.041 | 0.215 |
| *High* | -0.006 | -0.087 – 0.075 | 0.041 | 0.887 | -0.047 | -0.143 – 0.049 | 0.049 | 0.335 |
| Married | 0.044 | -0.026 – 0.113 | 0.035 | 0.215 | 0.011 | -0.051 – 0.074 | 0.032 | 0.717 |
| Income class:  [omitted = <500€] |  |  |  |  |  |  |  |  |
| *500€-1000€* | -0.022 | -0.124 – 0.080 | 0.052 | 0.668 | -0.011 | -0.113 – 0.091 | 0.052 | 0.833 |
| *1001€-1500€* | 0.003 | -0.089 – 0.095 | 0.047 | 0.953 | -0.030 | -0.125 – 0.065 | 0.048 | 0.535 |
| *1501€-2000€* | -0.028 | -0.128 – 0.072 | 0.051 | 0.581 | -0.053 | -0.157 – 0.051 | 0.053 | 0.318 |
| *2001€-2500€* | 0.069 | -0.073 – 0.211 | 0.072 | 0.341 | -0.002 | -0.119 – 0.115 | 0.059 | 0.975 |
| *2501€-3000€* | -0.079 | -0.210 – 0.052 | 0.067 | 0.235 | -0.077 | -0.213 – 0.058 | 0.069 | 0.262 |
| *>3000€* | -0.033 | -0.181 – 0.116 | 0.075 | 0.664 | -0.037 | -0.195 – 0.121 | 0.080 | 0.646 |
| *no answer* | -0.097 | -0.212 – 0.017 | 0.058 | 0.096 | -0.094 | -0.195 – 0.006 | 0.051 | 0.066 |
| Gift giving (pre-treatment):  [omitted = no gift] |  |  |  |  |  |  |  |  |
| *4€* | -0.221 | -0.379 – -0.063 | 0.080 | 0.006 | 0.063 | -0.068 – 0.195 | 0.067 | 0.344 |
| *8€* | -0.101 | -0.258 – 0.057 | 0.080 | 0.209 | 0.163 | 0.042 – 0.285 | 0.062 | 0.009 |
| *12€* | -0.022 | -0.175 – 0.130 | 0.078 | 0.774 | 0.124 | 0.023 – 0.224 | 0.051 | 0.016 |
| *16€* | -0.049 | -0.194 – 0.097 | 0.074 | 0.510 | 0.218 | 0.110 – 0.326 | 0.055 | 0.000 |
| *20€* | -0.055 | -0.196 – 0.087 | 0.072 | 0.446 | 0.201 | 0.102 – 0.300 | 0.050 | 0.000 |
| *24€* | 0.014 | -0.157 – 0.184 | 0.086 | 0.873 | 0.242 | 0.122 – 0.363 | 0.061 | 0.000 |
| *No answer* | -0.162 | -0.335 – 0.011 | 0.088 | 0.066 | -0.012 | -0.143 – 0.118 | 0.066 | 0.851 |
| Lenght of survey (ln) | 0.049 | -0.030 – 0.129 | 0.041 | 0.224 | -0.034 | -0.117 – 0.049 | 0.042 | 0.422 |

*Note.* CI = Confidence Intervals; SE = Standard Errors

**Table S6b. The effects of video and list conditions on trust by levels of *blatant* prejudice towards immigrants: linear model with robust SEs**

|  | *≤ median blatant prejudice towards immigrants* | | | | *> median blatant prejudice towards immigrants* | | | |
| --- | --- | --- | --- | --- | --- | --- | --- | --- |
|  | *b* | *95% CI(b)* | *SE(b)* | *p* | *b* | *95% CI(b)* | *SE(b)* | *p* |
| Intercept | 0.105 | -0.200 – 0.411 | 0.155 | 0.498 | 0.018 | -0.353 – 0.390 | 0.189 | 0.923 |
| Negative video | -0.016 | -0.137 – 0.106 | 0.062 | 0.800 | -0.028 | -0.142 – 0.086 | 0.058 | 0.630 |
| Positive video | 0.106 | -0.033 – 0.245 | 0.071 | 0.136 | -0.091 | -0.233 – 0.052 | 0.072 | 0.212 |
| Mixed list | 0.012 | -0.117 – 0.141 | 0.065 | 0.853 | -0.042 | -0.157 – 0.073 | 0.058 | 0.473 |
| Italian list | 0.026 | -0.089 – 0.141 | 0.058 | 0.659 | -0.044 | -0.172 – 0.084 | 0.065 | 0.502 |
| Negative video × Mixed list | 0.044 | -0.154 – 0.242 | 0.100 | 0.661 | 0.098 | -0.078 – 0.273 | 0.089 | 0.275 |
| Positive video × Mixed list | -0.101 | -0.292 – 0.090 | 0.097 | 0.298 | 0.134 | -0.057 – 0.325 | 0.097 | 0.169 |
| Negative video × Italian list | 0.043 | -0.132 – 0.217 | 0.089 | 0.632 | 0.140 | -0.040 – 0.320 | 0.091 | 0.126 |
| Positive video × Italian list | -0.145 | -0.330 – 0.041 | 0.094 | 0.126 | 0.089 | -0.099 – 0.276 | 0.095 | 0.353 |
| Age | -0.002 | -0.004 – 0.001 | 0.001 | 0.168 | 0.001 | -0.002 – 0.004 | 0.001 | 0.474 |
| Gender (being woman) | -0.076 | -0.137 – -0.014 | 0.031 | 0.016 | -0.012 | -0.078 – 0.053 | 0.033 | 0.708 |
| Education:  [omitted = Low] |  |  |  |  |  |  |  |  |
| *Middle* | 0.009 | -0.085 – 0.103 | 0.048 | 0.857 | 0.008 | -0.087 – 0.103 | 0.048 | 0.865 |
| *High* | 0.045 | -0.053 – 0.144 | 0.050 | 0.365 | 0.002 | -0.109 – 0.113 | 0.056 | 0.974 |
| Married | 0.043 | -0.031 – 0.117 | 0.038 | 0.253 | 0.057 | -0.009 – 0.123 | 0.034 | 0.090 |
| Income class:  [omitted = <500€] |  |  |  |  |  |  |  |  |
| *500€-1000€* | 0.019 | -0.106 – 0.144 | 0.063 | 0.765 | 0.046 | -0.052 – 0.144 | 0.050 | 0.357 |
| *1001€-1500€* | 0.002 | -0.096 – 0.100 | 0.050 | 0.968 | 0.047 | -0.063 – 0.157 | 0.056 | 0.400 |
| *1501€-2000€* | 0.002 | -0.112 – 0.115 | 0.058 | 0.973 | -0.033 | -0.148 – 0.082 | 0.058 | 0.577 |
| *2001€-2500€* | 0.058 | -0.096 – 0.212 | 0.078 | 0.459 | 0.017 | -0.124 – 0.159 | 0.072 | 0.810 |
| *2501€-3000€* | -0.016 | -0.212 – 0.179 | 0.099 | 0.870 | -0.018 | -0.170 – 0.135 | 0.077 | 0.821 |
| *>3000€* | -0.056 | -0.212 – 0.100 | 0.079 | 0.480 | 0.016 | -0.163 – 0.195 | 0.091 | 0.859 |
| *no answer* | -0.010 | -0.144 – 0.124 | 0.068 | 0.883 | -0.053 | -0.182 – 0.076 | 0.065 | 0.418 |
| Social preferences, pca (pre-treatment) | 0.025 | -0.000 – 0.050 | 0.013 | 0.050 | 0.025 | -0.002 – 0.052 | 0.014 | 0.074 |
| Lenght of survey (ln) | 0.137 | 0.044 – 0.230 | 0.047 | 0.004 | 0.101 | -0.008 – 0.210 | 0.055 | 0.068 |

*Note.* CI = Confidence Intervals; SE = Standard Errors

**Table S6c. The effects of video and list conditions on trustworthiness by levels of *blatant* prejudice towards immigrants: linear model with robust SEs**

|  | *≤ median blatant prejudice towards immigrants* | | | | *> median blatant prejudice towards immigrants* | | | |
| --- | --- | --- | --- | --- | --- | --- | --- | --- |
|  | *b* | *95% CI(b)* | *SE(b)* | *p* | *b* | *95% CI(b)* | *SE(b)* | *p* |
| Intercept | -0.173 | -0.268 – -0.077 | 0.048 | <0.001 | -0.071 | -0.203 – 0.061 | 0.067 | 0.292 |
| Trustor’s hypothetical transfer | 0.015 | 0.014 – 0.016 | 0.001 | <0.001 | 0.014 | 0.013 – 0.015 | 0.001 | <0.001 |
| Negative video | -0.016 | -0.059 – 0.027 | 0.022 | 0.460 | -0.015 | -0.054 – 0.023 | 0.020 | 0.439 |
| Positive video | 0.036 | -0.007 – 0.080 | 0.022 | 0.102 | 0.015 | -0.040 – 0.070 | 0.028 | 0.589 |
| Mixed list | -0.012 | -0.057 – 0.034 | 0.023 | 0.618 | 0.001 | -0.038 – 0.041 | 0.020 | 0.948 |
| Italian list | 0.004 | -0.043 – 0.051 | 0.024 | 0.862 | -0.025 | -0.062 – 0.012 | 0.019 | 0.192 |
| Negative video × Mixed list | -0.003 | -0.061 – 0.055 | 0.030 | 0.921 | 0.001 | -0.054 – 0.057 | 0.028 | 0.966 |
| Positive video × Mixed list | -0.032 | -0.093 – 0.030 | 0.031 | 0.310 | -0.025 | -0.095 – 0.044 | 0.035 | 0.470 |
| Negative video × Italian list | 0.042 | -0.023 – 0.107 | 0.033 | 0.209 | 0.061 | 0.007 – 0.115 | 0.027 | 0.027 |
| Positive video × Italian list | -0.019 | -0.083 – 0.045 | 0.033 | 0.557 | -0.004 | -0.075 – 0.067 | 0.036 | 0.910 |
| Age | -0.0004 | -0.001 – 0.001 | 0.0004 | 0.928 | -0.0004 | -0.001 – 0.0004 | 0.0004 | 0.285 |
| Gender (being woman) | -0.006 | -0.028 – 0.015 | 0.011 | 0.552 | -0.014 | -0.036 – 0.008 | 0.011 | 0.200 |
| Education:  [omitted = Low] |  |  |  |  |  |  |  |  |
| *Middle* | 0.015 | -0.028 – 0.059 | 0.022 | 0.485 | -0.009 | -0.042 – 0.023 | 0.017 | 0.571 |
| *High* | 0.018 | -0.024 – 0.061 | 0.021 | 0.393 | -0.001 | -0.039 – 0.038 | 0.019 | 0.978 |
| Married | 0.004 | -0.020 – 0.028 | 0.012 | 0.752 | 0.009 | -0.014 – 0.032 | 0.012 | 0.458 |
| Income class:  [omitted = <500€] |  |  |  |  |  |  |  |  |
| *500€-1000€* | 0.008 | -0.034 – 0.049 | 0.021 | 0.722 | 0.008 | -0.031 – 0.047 | 0.020 | 0.699 |
| *1001€-1500€* | 0.008 | -0.026 – 0.043 | 0.018 | 0.642 | -0.013 | -0.051 – 0.024 | 0.019 | 0.479 |
| *1501€-2000€* | -0.010 | -0.049 – 0.030 | 0.020 | 0.635 | 0.008 | -0.035 – 0.051 | 0.022 | 0.717 |
| *2001€-2500€* | 0.010 | -0.032 – 0.053 | 0.022 | 0.633 | -0.017 | -0.071 – 0.037 | 0.028 | 0.538 |
| *2501€-3000€* | -0.041 | -0.117 – 0.034 | 0.038 | 0.285 | -0.026 | -0.083 – 0.030 | 0.029 | 0.356 |
| *>3000€* | -0.006 | -0.064 – 0.051 | 0.029 | 0.826 | -0.023 | -0.080 – 0.035 | 0.029 | 0.440 |
| *no answer* | 0.016 | -0.023 – 0.055 | 0.020 | 0.425 | -0.030 | -0.066 – 0.006 | 0.018 | 0.099 |
| Social preferences, pca (pre-treatment) | 0.009 | 0.001 – 0.017 | 0.004 | 0.025 | 0.005 | -0.003 – 0.014 | 0.004 | 0.223 |
| Lenght of survey (ln) | 0.023 | -0.007 – 0.052 | 0.015 | 0.132 | 0.012 | -0.021 – 0.045 | 0.017 | 0.477 |

*Note.* CI = Confidence Intervals; SE = Standard Errors. Dependent variable: [Trustee’s return transfer / (3 x Trustor’s hypothetical transfer)]/100. The dataset is in the “long” format, i.e. each Trustee’s return decision is a distinct observation.

**Table S7a. The effects of video and list conditions on altruism by levels of *subtle* prejudice towards immigrants: linear model with robust SEs**

|  | *≤ median blatant prejudice towards immigrants* | | | | *> median blatant prejudice towards immigrants* | | | |
| --- | --- | --- | --- | --- | --- | --- | --- | --- |
|  | *b* | *95% CI(b)* | *SE(b)* | *p* | *b* | *95% CI(b)* | *SE(b)* | *p* |
| Intercept | 0.624 | 0.308 – 0.940 | 0.160 | 0.000 | 0.176 | -0.111 – 0.463 | 0.146 | 0.228 |
| Negative video | -0.002 | -0.125 – 0.121 | 0.062 | 0.974 | 0.080 | -0.040 – 0.200 | 0.061 | 0.192 |
| Positive video | 0.077 | -0.057 – 0.211 | 0.068 | 0.258 | 0.025 | -0.132 – 0.182 | 0.080 | 0.753 |
| Mixed list | -0.018 | -0.151 – 0.115 | 0.067 | 0.793 | 0.060 | -0.034 – 0.154 | 0.048 | 0.207 |
| Italian list | -0.043 | -0.162 – 0.077 | 0.061 | 0.481 | 0.029 | -0.078 – 0.135 | 0.054 | 0.593 |
| Negative video × Mixed list | 0.113 | -0.066 – 0.291 | 0.091 | 0.215 | -0.056 | -0.228 – 0.116 | 0.087 | 0.520 |
| Positive video × Mixed list | 0.019 | -0.156 – 0.194 | 0.089 | 0.830 | -0.055 | -0.253 – 0.143 | 0.100 | 0.586 |
| Negative video × Italian list | 0.084 | -0.099 – 0.266 | 0.092 | 0.367 | -0.117 | -0.289 – 0.056 | 0.088 | 0.184 |
| Positive video × Italian list | -0.053 | -0.250 – 0.145 | 0.100 | 0.600 | -0.121 | -0.310 – 0.068 | 0.096 | 0.210 |
| Age | -0.002 | -0.004 – 0.000 | 0.001 | 0.067 | 0.001 | -0.001 – 0.004 | 0.001 | 0.305 |
| Gender (being woman) | -0.051 | -0.110 – 0.008 | 0.030 | 0.090 | -0.035 | -0.091 – 0.021 | 0.028 | 0.220 |
| Education:  [omitted = Low] |  |  |  |  |  |  |  |  |
| *Middle* | -0.035 | -0.118 – 0.047 | 0.042 | 0.399 | -0.014 | -0.092 – 0.064 | 0.040 | 0.730 |
| *High* | -0.039 | -0.129 – 0.052 | 0.046 | 0.398 | 0.008 | -0.082 – 0.099 | 0.046 | 0.854 |
| Married | 0.067 | -0.005 – 0.139 | 0.037 | 0.068 | 0.015 | -0.046 – 0.076 | 0.031 | 0.625 |
| Income class:  [omitted = <500€] |  |  |  |  |  |  |  |  |
| *500€-1000€* | -0.017 | -0.119 – 0.085 | 0.052 | 0.744 | -0.020 | -0.123 – 0.082 | 0.052 | 0.694 |
| *1001€-1500€* | 0.005 | -0.090 – 0.101 | 0.049 | 0.912 | -0.046 | -0.139 – 0.046 | 0.047 | 0.327 |
| *1501€-2000€* | -0.056 | -0.160 – 0.049 | 0.053 | 0.294 | -0.028 | -0.138 – 0.083 | 0.056 | 0.622 |
| *2001€-2500€* | 0.011 | -0.114 – 0.137 | 0.064 | 0.861 | 0.034 | -0.102 – 0.170 | 0.069 | 0.619 |
| *2501€-3000€* | -0.181 | -0.323 – -0.038 | 0.072 | 0.013 | 0.014 | -0.101 – 0.129 | 0.059 | 0.811 |
| *>3000€* | -0.014 | -0.202 – 0.174 | 0.096 | 0.885 | -0.022 | -0.159 – 0.115 | 0.070 | 0.752 |
| *no answer* | -0.112 | -0.221 – -0.004 | 0.055 | 0.043 | -0.088 | -0.195 – 0.019 | 0.054 | 0.105 |
| Gift giving (pre-treatment):  [omitted = no gift] |  |  |  |  |  |  |  |  |
| *4€* | -0.213 | -0.386 – -0.040 | 0.088 | 0.016 | -0.012 | -0.152 – 0.127 | 0.071 | 0.860 |
| *8€* | -0.089 | -0.256 – 0.079 | 0.085 | 0.299 | 0.100 | -0.021 – 0.221 | 0.061 | 0.105 |
| *12€* | 0.010 | -0.146 – 0.165 | 0.079 | 0.904 | 0.072 | -0.038 – 0.181 | 0.056 | 0.201 |
| *16€* | -0.029 | -0.183 – 0.126 | 0.078 | 0.715 | 0.164 | 0.054 – 0.274 | 0.056 | 0.004 |
| *20€* | -0.006 | -0.154 – 0.142 | 0.075 | 0.937 | 0.129 | 0.015 – 0.244 | 0.058 | 0.027 |
| *24€* | 0.067 | -0.109 – 0.243 | 0.089 | 0.455 | 0.161 | 0.039 – 0.284 | 0.062 | 0.010 |
| *No answer* | -0.106 | -0.286 – 0.075 | 0.092 | 0.251 | -0.129 | -0.248 – -0.010 | 0.061 | 0.034 |
| Lenght of survey (ln) | -0.020 | -0.099 – 0.059 | 0.040 | 0.620 | 0.011 | -0.067 – 0.090 | 0.040 | 0.774 |

*Note.* CI = Confidence Intervals; SE = Standard Errors

**Table S7b. The effects of video and list conditions on trust by levels of *subtle* prejudice towards immigrants: linear model with robust SEs**

|  | *≤ median subtle prejudice towards immigrants* | | | | *> median subtle prejudice towards immigrants* | | | |
| --- | --- | --- | --- | --- | --- | --- | --- | --- |
|  | *b* | *95% CI(b)* | *SE(b)* | *p* | *b* | *95% CI(b)* | *SE(b)* | *p* |
| Intercept | 0.322 | 0.006 – 0.638 | 0.161 | 0.046 | -0.101 | -0.420 – 0.217 | 0.162 | 0.531 |
| Negative video | 0.023 | -0.101 – 0.148 | 0.063 | 0.712 | -0.049 | -0.163 – 0.065 | 0.058 | 0.402 |
| Positive video | 0.093 | -0.044 – 0.230 | 0.070 | 0.184 | -0.104 | -0.262 – 0.053 | 0.080 | 0.193 |
| Mixed list | -0.024 | -0.151 – 0.103 | 0.065 | 0.708 | 0.022 | -0.100 – 0.144 | 0.062 | 0.720 |
| Italian list | -0.024 | -0.161 – 0.113 | 0.070 | 0.731 | -0.004 | -0.114 – 0.105 | 0.056 | 0.936 |
| Negative video × Mixed list | 0.062 | -0.143 – 0.267 | 0.104 | 0.551 | 0.082 | -0.092 – 0.257 | 0.089 | 0.353 |
| Positive video × Mixed list | 0.051 | -0.144 – 0.246 | 0.099 | 0.606 | -0.034 | -0.230 – 0.161 | 0.099 | 0.731 |
| Negative video × Italian list | 0.086 | -0.091 – 0.262 | 0.090 | 0.342 | 0.084 | -0.105 – 0.274 | 0.096 | 0.380 |
| Positive video × Italian list | -0.079 | -0.276 – 0.119 | 0.100 | 0.435 | 0.028 | -0.169 – 0.224 | 0.100 | 0.782 |
| Age | -0.002 | -0.005 – 0.001 | 0.001 | 0.121 | -0.001 | -0.003 – 0.002 | 0.001 | 0.680 |
| Gender (being woman) | -0.046 | -0.108 – 0.016 | 0.032 | 0.148 | -0.014 | -0.078 – 0.050 | 0.032 | 0.660 |
| Education:  [omitted = Low] |  |  |  |  |  |  |  |  |
| *Middle* | 0.002 | -0.103 – 0.108 | 0.054 | 0.964 | 0.031 | -0.056 – 0.119 | 0.044 | 0.485 |
| *High* | 0.034 | -0.077 – 0.145 | 0.056 | 0.543 | 0.029 | -0.074 – 0.131 | 0.052 | 0.581 |
| Married | 0.093 | 0.018 – 0.168 | 0.038 | 0.015 | 0.035 | -0.031 – 0.102 | 0.034 | 0.293 |
| Income class:  [omitted = <500€] |  |  |  |  |  |  |  |  |
| *500€-1000€* | 0.051 | -0.063 – 0.165 | 0.058 | 0.379 | -0.006 | -0.115 – 0.104 | 0.056 | 0.919 |
| *1001€-1500€* | 0.003 | -0.094 – 0.100 | 0.049 | 0.956 | 0.011 | -0.107 – 0.129 | 0.060 | 0.853 |
| *1501€-2000€* | 0.006 | -0.104 – 0.115 | 0.056 | 0.918 | -0.022 | -0.145 – 0.101 | 0.062 | 0.720 |
| *2001€-2500€* | 0.092 | -0.048 – 0.232 | 0.071 | 0.195 | -0.029 | -0.179 – 0.120 | 0.076 | 0.701 |
| *2501€-3000€* | -0.048 | -0.227 – 0.131 | 0.091 | 0.597 | -0.005 | -0.162 – 0.153 | 0.080 | 0.955 |
| *>3000€* | -0.008 | -0.195 – 0.179 | 0.095 | 0.934 | 0.009 | -0.148 – 0.165 | 0.079 | 0.914 |
| *no answer* | -0.015 | -0.142 – 0.111 | 0.064 | 0.811 | -0.083 | -0.229 – 0.062 | 0.074 | 0.260 |
| Social preferences, pca (pre-treatment) | 0.038 | 0.015 – 0.061 | 0.012 | 0.001 | 0.011 | -0.014 – 0.036 | 0.013 | 0.383 |
| Lenght of survey (ln) | 0.051 | -0.041 – 0.144 | 0.047 | 0.272 | 0.174 | 0.076 – 0.273 | 0.050 | 0.001 |

*Note.* CI = Confidence Intervals; SE = Standard Errors

**Table S7c. The effects of video and list conditions on trustworthiness by levels of *subtle* prejudice towards immigrants: linear model with robust SEs**

|  | *≤ median subtle prejudice towards immigrants* | | | | *> median subtle prejudice towards immigrants* | | | |
| --- | --- | --- | --- | --- | --- | --- | --- | --- |
|  | *b* | *95% CI(b)* | *SE(b)* | *p* | *b* | *95% CI(b)* | *SE(b)* | *p* |
| Intercept | -0.062 | -0.174 – 0.050 | 0.057 | 0.276 | -0.177 | -0.299 – -0.056 | 0.062 | 0.004 |
| Trustor’s hypothetical transfer | 0.015 | 0.014 – 0.016 | 0.001 | <0.001 | 0.014 | 0.012 – 0.015 | 0.001 | <0.001 |
| Negative video | -0.007 | -0.049 – 0.035 | 0.021 | 0.751 | -0.017 | -0.055 – 0.021 | 0.019 | 0.377 |
| Positive video | 0.036 | -0.005 – 0.078 | 0.021 | 0.086 | 0.015 | -0.045 – 0.075 | 0.030 | 0.623 |
| Mixed list | -0.020 | -0.064 – 0.025 | 0.023 | 0.386 | 0.006 | -0.032 – 0.044 | 0.019 | 0.759 |
| Italian list | 0.009 | -0.037 – 0.054 | 0.023 | 0.711 | -0.020 | -0.057 – 0.018 | 0.019 | 0.298 |
| Negative video × Mixed list | 0.009 | -0.049 – 0.067 | 0.030 | 0.754 | -0.006 | -0.060 – 0.047 | 0.027 | 0.812 |
| Positive video × Mixed list | -0.008 | -0.067 – 0.050 | 0.030 | 0.782 | -0.046 | -0.120 – 0.028 | 0.038 | 0.225 |
| Negative video × Italian list | 0.022 | -0.037 – 0.082 | 0.030 | 0.460 | 0.066 | 0.010 – 0.122 | 0.028 | 0.022 |
| Positive video × Italian list | -0.041 | -0.108 – 0.025 | 0.034 | 0.223 | -0.001 | -0.074 – 0.072 | 0.037 | 0.987 |
| Age | -0.0002 | -0.001 – 0.001 | 0.0004 | 0.577 | -0.0005 | -0.001 – 0.0003 | 0.0004 | 0.199 |
| Gender (being woman) | -0.004 | -0.024 – 0.017 | 0.011 | 0.736 | -0.014 | -0.036 – 0.008 | 0.011 | 0.224 |
| Education:  [omitted = Low] |  |  |  |  |  |  |  |  |
| *Middle* | 0.017 | -0.023 – 0.056 | 0.020 | 0.405 | 0.000 | -0.036 – 0.037 | 0.019 | 0.988 |
| *High* | 0.021 | -0.018 – 0.060 | 0.020 | 0.294 | 0.006 | -0.033 – 0.046 | 0.020 | 0.750 |
| Married | 0.017 | -0.008 – 0.042 | 0.013 | 0.176 | 0.007 | -0.015 – 0.030 | 0.012 | 0.525 |
| Income class:  [omitted = <500€] |  |  |  |  |  |  |  |  |
| *500€-1000€* | 0.024 | -0.013 – 0.062 | 0.019 | 0.200 | -0.004 | -0.046 – 0.038 | 0.021 | 0.860 |
| *1001€-1500€* | 0.007 | -0.024 – 0.039 | 0.016 | 0.645 | -0.017 | -0.059 – 0.024 | 0.021 | 0.415 |
| *1501€-2000€* | -0.007 | -0.044 – 0.029 | 0.018 | 0.687 | 0.006 | -0.041 – 0.052 | 0.024 | 0.815 |
| *2001€-2500€* | 0.029 | -0.019 – 0.076 | 0.024 | 0.233 | -0.042 | -0.092 – 0.009 | 0.026 | 0.105 |
| *2501€-3000€* | -0.037 | -0.095 – 0.020 | 0.029 | 0.204 | -0.022 | -0.083 – 0.039 | 0.031 | 0.477 |
| *>3000€* | -0.007 | -0.075 – 0.062 | 0.035 | 0.845 | -0.018 | -0.074 – 0.038 | 0.028 | 0.522 |
| *no answer* | -0.000 | -0.035 – 0.035 | 0.018 | 0.992 | -0.024 | -0.067 – 0.018 | 0.021 | 0.260 |
| Social preferences, pca (pre-treatment) | 0.014 | 0.006 – 0.021 | 0.004 | 0.001 | 0.001 | -0.007 – 0.009 | 0.004 | 0.807 |
| Lenght of survey (ln) | -0.015 | -0.046 – 0.015 | 0.016 | 0.329 | 0.048 | 0.016 – 0.080 | 0.016 | 0.003 |

*Note.* CI = Confidence Intervals; SE = Standard Errors. Dependent variable: [Trustee’s return transfer / (3 x Trustor’s hypothetical transfer)]/100. The dataset is in the “long” format, i.e. each Trustee’s return decision is a distinct observation.

**Table S8. The effects of video and list conditions on intergroup anxiety: linear model with robust SEs**

|  | *b* | *95% CI(b)* | *SE(b)* | *p* |
| --- | --- | --- | --- | --- |
| Intercept | 2.667 | 1.212 – 4.122 | 0.741 | < 0.001 |
| Negative video | 0.688 | 0.135 – 1.241 | 0.282 | 0.015 |
| Positive video | 0.160 | -0.497 – 0.817 | 0.334 | 0.632 |
| Mixed list | 0.149 | -0.470 – 0.769 | 0.315 | 0.636 |
| Italian list | 0.044 | -0.585 – 0.674 | 0.320 | 0.890 |
| Negative video × Mixed list | -0.444 | -1.375 – 0.487 | 0.474 | 0.349 |
| Positive video × Mixed list | 0.249 | -0.635 – 1.134 | 0.450 | 0.580 |
| Negative video × Italian list | -0.843 | -1.713 – 0.028 | 0.443 | 0.058 |
| Positive video × Italian list | -0.160 | -1.120 – 0.800 | 0.489 | 0.744 |
| Age | -0.015 | -0.027 – -0.003 | 0.006 | 0.017 |
| Gender (being woman) | 0.202 | -0.124 – 0.528 | 0.166 | 0.224 |
| Married | -0.157 | -0.504 – 0.190 | 0.177 | 0.375 |
| Education:  [omitted = Low] |  |  |  |  |
| *Middle* | 0.020 | -0.495 – 0.534 | 0.262 | 0.940 |
| *High* | -0.013 | -0.541 – 0.514 | 0.268 | 0.960 |
| Income class:  [omitted = <500€] |  |  |  |  |
| *500€-1000€* | -0.358 | -0.993 – 0.276 | 0.323 | 0.268 |
| *1001€-1500€* | -0.644 | -1.236 – -0.052 | 0.301 | 0.033 |
| *1501€-2000€* | -0.290 | -0.899 – 0.319 | 0.310 | 0.350 |
| *2001€-2500€* | -0.210 | -0.919 – 0.499 | 0.361 | 0.561 |
| *2501€-3000€* | 0.129 | -0.858 – 1.117 | 0.503 | 0.797 |
| *>3000€* | -0.513 | -1.307 – 0.280 | 0.404 | 0.204 |
| *no answer* | -0.322 | -0.965 – 0.321 | 0.327 | 0.326 |
| Anti-immigration attitudes, pca (pre-treatment) | 0.289 | 0.224 – 0.354 | 0.033 | < 0.001 |
| Lenght of survey (ln) | -0.603 | -1.035 – -0.170 | 0.220 | 0.006 |

*Note.* CI = Confidence Intervals; SE = Standard Errors.

**Table S9. The effects of video and list conditions on perceived outgroup threat: linear model with robust SEs**

|  | *b* | *95% CI(b)* | *SE(b)* | *p* |
| --- | --- | --- | --- | --- |
| Intercept | 0.561 | -0.517 – 1.638 | 0.548 | 0.307 |
| Negative video | 0.627 | 0.255 – 0.998 | 0.189 | 0.001 |
| Positive video | 0.142 | -0.265 – 0.549 | 0.207 | 0.493 |
| Mixed list | -0.124 | -0.500 – 0.252 | 0.191 | 0.516 |
| Italian list | 0.109 | -0.281 – 0.500 | 0.199 | 0.582 |
| Negative video × Mixed list | -0.116 | -0.697 – 0.465 | 0.296 | 0.695 |
| Positive video × Mixed list | 0.237 | -0.337 – 0.812 | 0.292 | 0.417 |
| Negative video × Italian list | -0.681 | -1.261 – -0.102 | 0.295 | 0.021 |
| Positive video × Italian list | -0.308 | -0.898 – 0.282 | 0.300 | 0.305 |
| Age | 0.005 | -0.003 – 0.013 | 0.004 | 0.209 |
| Gender (being woman) | -0.050 | -0.257 – 0.158 | 0.106 | 0.639 |
| Married | -0.038 | -0.249 – 0.173 | 0.108 | 0.723 |
| Education:  [omitted = Low] |  |  |  |  |
| *Middle* | -0.178 | -0.538 – 0.181 | 0.183 | 0.329 |
| *High* | -0.195 | -0.587 – 0.196 | 0.199 | 0.328 |
| Income class:  [omitted = <500€] |  |  |  |  |
| *500€-1000€* | -0.078 | -0.459 – 0.302 | 0.194 | 0.686 |
| *1001€-1500€* | -0.172 | -0.540 – 0.195 | 0.187 | 0.358 |
| *1501€-2000€* | 0.007 | -0.370 – 0.384 | 0.192 | 0.971 |
| *2001€-2500€* | -0.172 | -0.620 – 0.276 | 0.228 | 0.450 |
| *2501€-3000€* | 0.266 | -0.331 – 0.864 | 0.304 | 0.382 |
| *>3000€* | -0.317 | -0.980 – 0.345 | 0.337 | 0.347 |
| *no answer* | -0.014 | -0.431 – 0.402 | 0.212 | 0.946 |
| Anti-immigration attitudes, pca (pre-treatment) | 0.614 | 0.578 – 0.651 | 0.018 | < 0.001 |
| Lenght of survey (ln) | -0.210 | -0.513 – 0.093 | 0.154 | 0.173 |

*Note.* CI = Confidence Intervals; SE = Standard Errors.

**Table S10. The effects of video and list conditions on perceived health risk: linear model with robust SEs**

|  | *b* | *95% CI(b)* | *SE(b)* | *p* |
| --- | --- | --- | --- | --- |
| Intercept | -0.600 | -2.006 – 0.806 | 0.716 | 0.402 |
| Negative video | 0.649 | 0.098 – 1.201 | 0.281 | 0.021 |
| Positive video | 0.297 | -0.244 – 0.838 | 0.275 | 0.282 |
| Mixed list | -0.086 | -0.609 – 0.437 | 0.266 | 0.746 |
| Italian list | 0.246 | -0.257 – 0.750 | 0.256 | 0.337 |
| Negative video × Mixed list | -0.180 | -0.991 – 0.632 | 0.413 | 0.664 |
| Positive video × Mixed list | 0.374 | -0.359 – 1.106 | 0.373 | 0.317 |
| Negative video × Italian list | -0.952 | -1.701 – -0.203 | 0.381 | 0.013 |
| Positive video × Italian list | -0.509 | -1.249 – 0.232 | 0.377 | 0.178 |
| Age | -0.001 | -0.011 – 0.009 | 0.005 | 0.892 |
| Gender (being woman) | 0.162 | -0.094 – 0.419 | 0.130 | 0.214 |
| Married | -0.102 | -0.388 – 0.184 | 0.146 | 0.485 |
| Education:  [omitted = Low] |  |  |  |  |
| *Middle* | 0.040 | -0.420 – 0.501 | 0.234 | 0.863 |
| *High* | 0.006 | -0.490 – 0.502 | 0.253 | 0.982 |
| Income class:  [omitted = <500€] |  |  |  |  |
| *500€-1000€* | -0.088 | -0.609 – 0.433 | 0.265 | 0.741 |
| *1001€-1500€* | -0.128 | -0.627 – 0.371 | 0.254 | 0.615 |
| *1501€-2000€* | -0.180 | -0.698 – 0.337 | 0.263 | 0.494 |
| *2001€-2500€* | 0.012 | -0.649 – 0.672 | 0.336 | 0.972 |
| *2501€-3000€* | 0.038 | -0.672 – 0.749 | 0.361 | 0.915 |
| *>3000€* | -0.048 | -0.706 – 0.610 | 0.335 | 0.886 |
| *no answer* | 0.121 | -0.457 – 0.700 | 0.295 | 0.680 |
| Anti-immigration attitudes, pca (pre-treatment) | 0.576 | 0.527 – 0.624 | 0.025 | < 0.001 |
| Lenght of survey (ln) | 0.134 | -0.233 – 0.501 | 0.187 | 0.473 |

*Note.* CI = Confidence Intervals; SE = Standard Errors.

**Figure S3.** The effects of media portrayals of immigrants on outgroup-related perceived health risk (Study 2)


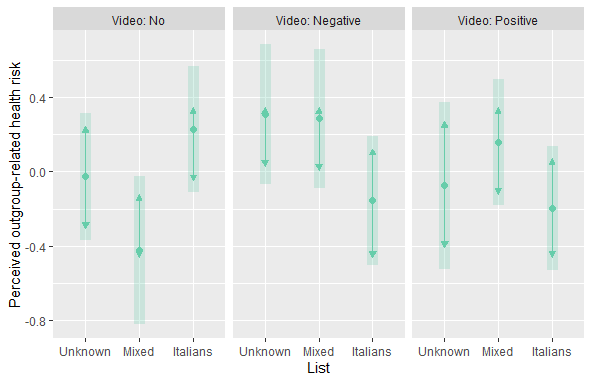


Estimated marginal means (range 0-1) of by video and list conditions (Tukey adjustment; bars: 95% confidence intervals; non-overlapping arrows: statistically significant pairwise comparison, *p* < .05). Sample: Study 2 participants.

**Additional materials (SM2)**

1. **Pre-test of video conditions on a separate, independent sample of university students**

1. **Perceived valence of the videos - respondents in Study 1.**

1. **Perceived valence of the videos - respondents in Study 2.**

1. **Subjective probability of playing with an immigrant partner**

*Note.* Colored bars report standardized respondents’ subjective probabilities of playing with an immigrant participant, as elicited through the post-treatments question: “Indicate with what probability, from 0% to 100%, you think you played with i) An Italian player:__; ii) An immigrant player: __”; confidence intervals are shown as gray lines above the bars.

1. **The effects of video and list conditions on risk tolerance: ordered logit model with robust SEs**

*STUDY 1*

|  | *b* | *95% CI(b)* | *SE(b)* | *p* |
| --- | --- | --- | --- | --- |
| Age | -0.006 | -0.051 – 0.040 | 0.023 | 0.807 |
| Gender (being woman) | -0.476 | -0.881 – -0.071 | 0.207 | 0.021 |
| Negative video | -0.347 | -1.033 – 0.340 | 0.350 | 0.322 |
| Positive video | -0.318 | -1.038 – 0.402 | 0.367 | 0.386 |
| Italian list | -0.091 | -0.852 – 0.670 | 0.388 | 0.815 |
| Mixed list | 0.085 | -0.717 – 0.887 | 0.409 | 0.835 |
| Negative video × Mixed list | 0.596 | -0.466 – 1.658 | 0.542 | 0.271 |
| Positive video × Mixed list | -0.107 | -1.198 – 0.984 | 0.557 | 0.848 |
| Negative video × Italian list | 0.049 | -1.088 – 1.186 | 0.580 | 0.933 |
| Positive video × Italian list | 0.147 | -0.973 – 1.268 | 0.572 | 0.797 |

*Note.* CI = Confidence Intervals; SE = Standard Errors.

*STUDY 2*

|  | *b* | *95% CI(b)* | *SE(b)* | *p* |
| --- | --- | --- | --- | --- |
| Negative video | 0.248 | -0.363 – 0.859 | 0.312 | 0.427 |
| Positive video | 0.555 | -0.333 – 1.444 | 0.453 | 0.220 |
| Italian list | 0.161 | -0.492 – 0.815 | 0.333 | 0.628 |
| Mixed list | 0.184 | -0.406 – 0.773 | 0.301 | 0.541 |
| Negative video × Mixed list | 0.004 | -0.897 – 0.905 | 0.460 | 0.993 |
| Positive video × Mixed list | -0.190 | -1.258 – 0.877 | 0.545 | 0.727 |
| Negative video × Italian list | 0.008 | -0.937 – 0.953 | 0.482 | 0.987 |
| Positive video × Italian list | -0.499 | -1.663 – 0.664 | 0.594 | 0.400 |
| Age | -0.002 | -0.016 – 0.011 | 0.007 | 0.743 |
| Gender (being woman) | 0.076 | -0.269 – 0.420 | 0.176 | 0.667 |
| Married | 0.349 | -0.013 – 0.712 | 0.185 | 0.059 |
| Education:  [omitted = Low] |  |  |  |  |
| *Middle* | -0.503 | -1.022 – 0.016 | 0.265 | 0.057 |
| *High* | -0.726 | -1.269 – -0.183 | 0.277 | 0.009 |
| Income class:  [omitted = <500€] |  |  |  |  |
| *500€-1000€* | 0.279 | -0.330 – 0.888 | 0.311 | 0.369 |
| *1001€-1500€* | 0.378 | -0.153 – 0.908 | 0.271 | 0.163 |
| *1501€-2000€* | 0.112 | -0.504 – 0.727 | 0.314 | 0.722 |
| *2001€-2500€* | 0.315 | -0.387 – 1.016 | 0.358 | 0.380 |
| *2501€-3000€* | 1.125 | 0.047 – 2.203 | 0.550 | 0.041 |
| *>3000€* | -0.041 | -0.873 – 0.790 | 0.424 | 0.923 |
| *no answer* | 0.241 | -0.377 – 0.859 | 0.315 | 0.446 |
| Risk attitudes (pre-treatment) | -0.007 | -0.114 – 0.099 | 0.054 | 0.893 |
| Lenght of survey (ln) | -0.438 | -0.878 – 0.001 | 0.224 | 0.051 |

*Note.* CI = Confidence Intervals; SE = Standard Errors.

**Supplementary materials 3 (SM3)**

***** Replication of Study 1 results clustering standard errors at the session level *****

**Table A2. The effects of video and list conditions on altruism: linear model with robust SEs**

|  | *b* | *95% CI(b)* | *SE(b)* | *p* |
| --- | --- | --- | --- | --- |
| Intercept | 0.387 | 0.22 – 0.55 | 0.078 | < 0.001 |
| Age | 0.004 | -0.00 – 0.01 | 0.003 | 0.225 |
| Gender (being woman) | -0.049 | -0.12 – 0.02 | 0.034 | 0.173 |
| Negative video | -0.112 | -0.17 – -0.05 | 0.029 | 0.002 |
| Positive video | -0.007 | -0.18 – 0.17 | 0.082 | 0.928 |
| Mixed list | -0.041 | -0.09 – 0.01 | 0.024 | 0.109 |
| Italian list | -0.074 | -0.13 – -0.01 | 0.028 | 0.018 |
| Negative video × Mixed list | 0.011 | -0.05 – 0.07 | 0.028 | 0.709 |
| Positive video × Mixed list | -0.026 | -0.20 – 0.15 | 0.083 | 0.760 |
| Negative video × Italian list | 0.188 | 0.10 – 0.28 | 0.042 | < 0.001 |
| Positive video × Italian list | -0.033 | -0.21 – 0.14 | 0.081 | 0.694 |

*Note.* Standard Errors clustered at the session level. CI = Confidence Intervals; SE = Standard Errors.

**Table A3. The effects of video and list conditions on trust: linear model with robust SEs**

|  | *b* | *95% CI(b)* | *SE(b)* | *p* |
| --- | --- | --- | --- | --- |
| Intercept | 0.598 | 0.41 – 0.78 | 0.087 | < 0.001 |
| Age | 0.000 | -0.01– 0.01 | 0.004 | 0.935 |
| Gender (being woman) | -0.114 | -0.18 – -0.05 | 0.030 | 0.002 |
| Negative video | -0.018 | -0.06 – 0.03 | 0.022 | 0.433 |
| Positive video | -0.034 | -0.11 – 0.04 | 0.034 | 0.328 |
| Mixed list | -0.060 | -0.13 – 0.01 | 0.035 | 0.108 |
| Italian list | -0.095 | -0.20 – 0.00 | 0.047 | 0.061 |
| Negative video × Mixed list | 0.036 | -0.06 – 0.13 | 0.045 | 0.430 |
| Positive video × Mixed list | 0.085 | -0.08 – 0.25 | 0.080 | 0.304 |
| Negative video × Italian list | 0.093 | -0.01 – 0.20 | 0.048 | 0.069 |
| Positive video × Italian list | 0.072 | -0.06 – 0.20 | 0.060 | 0.248 |

*Note.* Standard Errors clustered at the session level. CI = Confidence Intervals; SE = Standard Errors.

**Table A4. The effects of video and list conditions on trustworthiness (full set of strategies): linear model with robust SEs**

|  | *b* | *95% CI(b)* | *SE(b)* | *p* |
| --- | --- | --- | --- | --- |
| Intercept | 1.356 | 0.954 – 1.757 | 0.188 | 0.000 |
| Trustor’s hypothetical transfer | 0.010 | 0.001 – 0.019 | 0.004 | 0.038 |
| Age | 0.004 | -0.011 – 0.019 | 0.007 | 0.596 |
| Gender (being woman) | -0.013 | -0.168 – 0.141 | 0.072 | 0.856 |
| Negative video | -0.229 | -0.494 – 0.035 | 0.124 | 0.084 |
| Positive video | -0.082 | -0.312 – 0.148 | 0.108 | 0.458 |
| Mixed list | -0.105 | -0.381 – 0.172 | 0.130 | 0.432 |
| Italian list | -0.034 | -0.198 – 0.131 | 0.077 | 0.668 |
| Negative video × Mixed list | 0.195 | -0.197 – 0.587 | 0.184 | 0.306 |
| Positive video × Mixed list | 0.100 | -0.223 – 0.423 | 0.151 | 0.520 |
| Negative video × Italian list | 0.301 | 0.021 – 0.582 | 0.131 | 0.037 |
| Positive video × Italian list | -0.062 | -0.354 – 0.229 | 0.137 | 0.656 |

*Note.* Standard Errors clustered at the session level. CI = Confidence Intervals; SE = Standard Errors. Dependent variable: Trustee’s return transfer / Trustor’s hypothetical transfer. The dataset is in the “long” format, i.e. each Trustee’s return decision is a distinct observation (N = 3,468).

**Table A5. The effects of video conditions on growth rate of testosterone-cortisol ratio: linear model with robust SEs**

|  | *b* | *95% CI(b)* | *SE(b)* | *p* |
| --- | --- | --- | --- | --- |
| Intercept | 0.590 | 0.13 – 1.05 | 0.216 | 0.015 |
| Age | -0.001 | -0.02 – 0.01 | 0.007 | 0.915 |
| Gender (being woman) | -0.306 | -0.45 – -0.16 | 0.067 | < 0.001 |
| Baseline level | -0.001 | -0.00 – -0.00 | 0.000 | 0.001 |
| Negative video | 0.385 | 0.20 – 0.57 | 0.085 | < 0.001 |
| Positive video | 0.072 | -0.11 – 0.25 | 0.084 | 0.410 |

*Note.* Standard Errors clustered at the session level. CI = Confidence Intervals; SE = Standard Errors.

**Table A6. The effects of video conditions on intergroup anxiety: linear model with robust SEs**

|  | *b* | *95% CI(b)* | *SE(b)* | *p* |
| --- | --- | --- | --- | --- |
| Intercept | 0.130 | -1.120 – 1.379 | 0.586 | 0.828 |
| Age | -0.013 | -0.057 – 0.030 | 0.020 | 0.519 |
| Gender (being woman) | 0.241 | -0.133 – 0.614 | 0.175 | 0.190 |
| Negative video | 0.272 | -0.316 – 0.859 | 0.276 | 0.340 |
| Positive video | -0.164 | -0.669 – 0.342 | 0.237 | 0.501 |

*Note.* Standard Errors clustered at the session level. CI = Confidence Intervals; SE = Standard Errors.

**Table A7. The effects of video conditions on perceived outgroup threat: linear model with robust SEs**

|  | *b* | *95% CI(b)* | *SE(b)* | *p* |
| --- | --- | --- | --- | --- |
| Intercept | 0.377 | -0.537 – 1.291 | 0.429 | 0.393 |
| Age | -0.012 | -0.047 – 0.023 | 0.016 | 0.464 |
| Gender (being woman) | -0.540 | -0.915 – -0.166 | 0.176 | 0.008 |
| Negative video | 0.546 | 0.362 – 0.731 | 0.087 | < 0.001 |
| Positive video | 0.210 | -0.145 – 0.566 | 0.167 | 0.226 |

*Note.* Standard Errors clustered at the session level. CI = Confidence Intervals; SE = Standard Errors

**Figure A1. The effects of media portrayals of immigrants on prosociality (Study 1)**

**PANEL A**


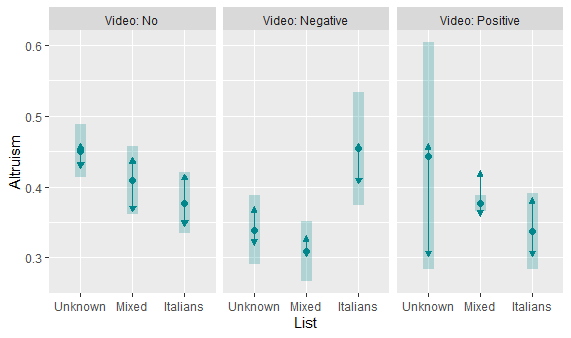


*p* < 0.001

*p* = 0.004

**PANEL B**


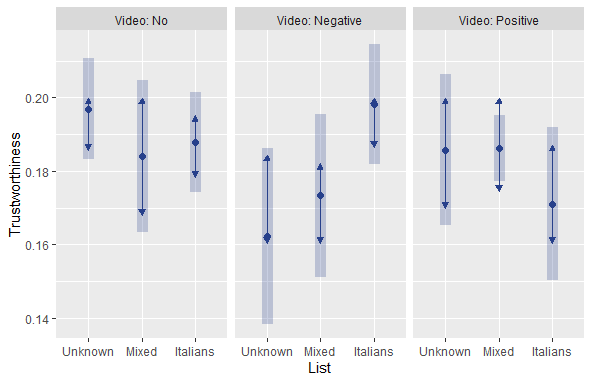


*p* = 0.034

*p* = 0.012

Estimated marginal means (range 0-1) of altruism (PANEL A) and trustworthiness (PANEL B) by video and list conditions (Tukey adjustment; bars: 95% confidence intervals; non-overlapping arrows: statistically significant pairwise comparison, *p* < .05). Altruism: money sent to the partner over initial endowment in dictator game; Trustworthiness: money returned over received in trust game, i.e. [Trustee’s return transfer / (3 x Trustor’s hypothetical transfer)]/100; the dataset is in the “long” format, i.e. each Trustee’s return decision is a distinct observation. Sample: Study 1 participants.

**Figure A2. The physiological effects of media portrayals of immigrants (Study 1)**


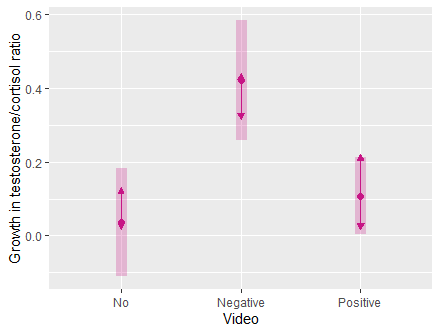


*p* = 0.003

*p* < 0.001

Estimated marginal means (range 0-1) of testosterone-cortisol ratio growth rate by video conditions (Tukey adjustment; bars: 95% confidence intervals; non-overlapping arrows: statistically significant pairwise comparison, *p* < .05). Sample: Study 1 participants.
